# Musical training shapes neural responses to melodic and prosodic expectation

**DOI:** 10.1016/j.brainres.2016.09.015

**Published:** 2016-11-01

**Authors:** Ioanna Zioga, Caroline Di Bernardi Luft, Joydeep Bhattacharya

**Affiliations:** a Department of Psychology, Goldsmiths, University of London, New Cross, London SE14 6NW, United Kingdom; b School of Biological and Chemical Sciences, Queen Mary, University of London, Mile End Rd, London E1 4NS, United Kingdom

**Keywords:** EEG, Expectation, Musical training, Language, Prosody

## Abstract

Current research on music processing and syntax or semantics in language suggests that music and language share partially overlapping neural resources. Pitch also constitutes a common denominator, forming melody in music and prosody in language. Further, pitch perception is modulated by musical training. The present study investigated how music and language interact on pitch dimension and whether musical training plays a role in this interaction. For this purpose, we used melodies ending on an expected or unexpected note (melodic expectancy being estimated by a computational model) paired with prosodic utterances which were either expected (statements with falling pitch) or relatively unexpected (questions with rising pitch). Participants' (22 musicians, 20 nonmusicians) ERPs and behavioural responses in a statement/question discrimination task were recorded. Participants were faster for simultaneous expectancy violations in the melodic and linguistic stimuli. Further, musicians performed better than nonmusicians, which may be related to their increased pitch tracking ability. At the neural level, prosodic violations elicited a front-central positive ERP around 150 ms after the onset of the last word/note, while musicians presented reduced P600 in response to strong incongruities (questions on low-probability notes). Critically, musicians' P800 amplitudes were proportional to their level of musical training, suggesting that expertise might shape the pitch processing of language. The beneficial aspect of expertise could be attributed to its strengthening effect of general executive functions. These findings offer novel contributions to our understanding of shared higher-order mechanisms between music and language processing on pitch dimension*,* and further demonstrate a potential modulation by musical expertise.

## Highlights

• Melodic expectancy influences the processing of prosodic expectancy.
• Musical expertise modulates pitch processing in music and language.
• Musicians have a more refined response to pitch.
• Musicians' neural responses are proportional to their level of musical expertise.
• Possible association between the P200 neural component and behavioural facilitation.

## 1 Introduction

Music and language are two of the most characteristic human attributes, and there has been a surge of recent research interest in investigating the relationship between their cognitive and neural processing (e.g., Carrus et al., 2011; Koelsch and Jentschke, 2010; Maess et al., 2001; Patel et al., 1998a, Patel et al., 1998b). Music and language use different elements (i.e. tones and words, respectively) to form complex hierarchical structures (i.e. harmony and sentences, respectively), governed by a set of rules which determines their syntax (Patel, 1998, Patel, 2003, 2012; Slevc et al., 2009). However, analogies between the two domains should be done carefully, as grammatical categories (nouns, verbs) and functions (subject, object) have no parallels in music (Jackendoff, 2009, Patel, 2003). Further, musical elements can be played concurrently to form harmony, but this is not the case for language.

In this context, Patel (1998) hypothesised that what is common in music and language is that experienced listeners organise their elements in an hierarchical fashion based on learned rules (McMullen and Saffran, 2004, Slevc et al., 2009). Importantly, through everyday exposure to these rules, expectations for subsequent events are formed (Jonaitis and Saffran, 2009; Meyer, 2008). The fulfilment or violation of expectations constitutes a crucial component of the emotional and aesthetic experience of music (Huron, 2006). Expectations can also be disrupted in language, resulting in unexpected or incorrect sentences (Gibson, 2006).

Based upon their structural similarities, theoretical work has suggested that music and language use overlapping neural resources (Patel, 2003, Patel et al., 1998a, Patel et al., 1998b). In his *‘Shared Syntactic Integration Resource Hypothesis’* (SSIRH) Patel (2003) has suggested that shared domain-general neural areas in frontal regions would process syntactic information, which would be then integrated at posterior domain-specific representational sites (Fedorenko et al., 2009; Koelsch, 2012; Patel, 2008). Indeed, it has been shown that music influences simultaneous responses to language, due to competition for shared resources between harmony (chord sequences) and syntax (Carrus et al., 2013, Fedorenko et al., 2009, Jung et al., 2015; Koelsch et al., 2005; Kunert et al., 2015; Patel, 2003; Perruchet and Poulin-Charronnat, 2013; Slevc et al., 2009). For example, Carrus et al. (2013) found that harmonically unexpected notes influence neural responses to linguistic syntax, but not to semantics. Furthermore, studies have shown that sentence comprehension declines when an incongruent word co-occurs with an out-of-key note (Fedorenko et al., 2009, Slevc et al., 2009), providing behavioural evidence for interactions between the two domains. These interactions might provide evidence for shared, partially overlapping neural resources involved in the processing of syntax in language and music (e.g., Carrus et al., 2013; Koelsch et al., 2005). Critically, the present study did not investigate these aspects; rather we manipulated melodic and prosodic features to investigate potential shared processes in pitch dimension.

In fact, besides harmony and syntax, pitch is another important feature, forming the melody in music and the intonation or prosody (*‘the melody of speech’*) in language. Prosody is not only limited to fundamental frequency (*F*_*0*_) fluctuations, but also refers to other properties of speech, such as fluctuations of loudness, stretching and shrinking of segments and syllable durations, speech rate and voice quality (Ashby and Maidment, 2005, Chun, 2002, Hirst and Cristo, 1998, Nolan, 2008, Nooteboom, 1997, Selkirk, 1995, Wells, 2006). Prosodic cues are used during on-line sentence comprehension to establish the syntactic structure and provide semantic information (Holzgrefe et al., 2013, Kotz et al., 2003, Steinhauer et al., 1999). Further, prosody serves communicative functions, as it allows to differentiate speech acts as questions or declaratives, and infers emotions (Pannekamp and Toepel, 2005, Paulmann et al., 2012).

In intonation languages, such as English and German, final tones are usually falling in pitch in statements (Meyer et al., 2002). The intonation contour of questions depends on the type of interrogation. Four main types of questions have been identified: alternative questions (e.g., *“Is he alright or not? ”*), yes-no questions (e.g., *“Is he alright? ”*), wh-questions (e.g., *“Who is he? ”*), and declarative questions ( or ‘non-interrogative’) (e.g., *“He is alright? ”*) (Bartels, 1997, Bartels, 1999, Grabe, 2004). The rise of final intonation contour is significantly more common than low contour in all dialects, for yes-no questions, wh-questions, and declarative questions (Grabe, 2004). The exception is for wh-questions, where falling final pitch is more usual, as the more the lexical markers of interrogativity there are in an utterance, the less the final pitch rises (wh-questions have two markers: the initial “wh-” and the word inversion) (Haan, 2002). In our study, we used statements, and declarative questions with a final rise in the intonation contour.

In order to investigate potential effects of melodic expectancy on prosodic processing, our participants were asked to pay attention only to the speech while ignoring the music. Previous research has demonstrated qualitative differences between early vs. late ERP components, suggesting that early ERPs reflect sensory and perceptual processing modulated by attention (Hillyard et al., 1973, Näätänen et al., 1978), whereas late ERPs reflect integration and re-analysis processes (e.g., Astésano et al., 2004; Eckstein and Friederici, 2005). Studies using auditory stimuli have shown that N1 is enhanced when attention is directed to a stimulus (Alho et al., 1994, Schirmer and Kotz, 2006), as well as in response to unexpected events (Debener et al., 2005, Proverbio et al., 2004). In addition, musicians show larger P200 component, which is attributed to neuroplastic effects of musical expertise (Shahin et al., 2003, Trainor et al., 2003).

ERP responses to melodic and prosodic violation of expectations have been investigated separately in music and language (e.g., Astésano et al., 2004; Besson and Faïta, 1995; Koelsch and Friederici, 2003; Koelsch and Jentschke, 2010; Lindsen et al., 2010; Patel et al., 1998a; Paulmann et al., 2012). It has been found that melodically unexpected notes elicit late positive components (LPCs) with a parietal scalp distribution around 300 ms post-stimulus onset (larger amplitude and shorter latency for non-diatonic compared to diatonic incongruities) (Besson and Faïta, 1995). Prosodic expectancy violations in speech utterances have been found to elicit a late positive component (*‘prosodic expectancy positivity’* or PEP) (Paulmann et al., 2012, Paulmann and Kotz, 2008), as well as a task-dependent, left temporo-parietal positivity (P800) (Astésano et al., 2004), associated with prosodic reanalysis processes. More specifically, when the prosody cannot be mapped onto the syntactic structure, the listener has to reanalyse the sentence in order to make sense of it (e.g., the utterance *Sarah is having lunch at the restaurant?* has unexpected prosody, as its syntax guides us to perceive it as a statement until the question is formed at the end). The P600 component has also been associated with violation of prosodic expectancy or prosody-syntax mismatch, reflecting structural revision processes (Eckstein and Friederici, 2005; Friederici and Alter, 2004; Schön et al., 2002; Steinhauer et al., 1999). Confirming the aforementioned findings, an fMRI study revealed increased BOLD activity in bilateral inferior frontal and temporal regions for unexpected compared to expected prosodic utterances (Doherty et al., 2004).

There is evidence that musical training alters the nervous system (Ragert et al., 2004), as well as the sensitivity to different aspects of musical sounds (Habib and Besson, 2009). Indeed, long-term musical training has been found to enhance brainstem responses to musical pitch (Skoe and Kraus, 2012), while musicians’ auditory processing benefits have been positively correlated with the amount of musical expertise and the age that musical training started (Zendel and Alain, 2013). Supporting the view of shared processing, there is evidence for bidirectional influences between musical training and language skills (Asaridou and McQueen, 2013, Moreno, 2009, Schön et al., 2004, Wong et al., 2007, Zhao and Kuhl, 2015). For example, music experience has been shown to improve reading abilities in young children (Anvari et al., 2002). Musically trained listeners have shown better performance in detecting deviations of speech intonation (Schön et al., 2004), as well as interpreting affective prosody compared to untrained listeners (Thompson et al., 2004). Native English musicians have shown superior brainstem encoding of linguistic pitch patterns in a study using Mandarin Chinese syllables compared to nonmusicians (Wong et al., 2007). Interestingly, there is also evidence for the opposite effect (e.g., Bidelman et al., 2011; Bradley, 2012). Specifically, speakers of Mandarin have been shown to perform better than English speakers in detection of pitch changes (Bradley, 2012). Thus, it seems that experience in one domain can be beneficial for the other one, suggesting that pitch tracking ability might be a potential shared mechanism (Asaridou and McQueen, 2013, Bidelman et al., 2011).

However, as both music performance and linguistic efficiency demand high levels of cognitive control, research has suggested that the aforementioned bidirectional influences could be attributed to enhanced executive functions due to musical training (Bialystok and Depape, 2009, Ho et al., 2003, Moreno et al., 2011, Schellenberg, 2004, Schellenberg, 2003, Schellenberg, 2006) or bilingualism (e.g., Bialystok and Viswanathan, 2009; Festman et al., 2010; Krizman et al., 2012; Poarch and van Hell, 2012). For example, there is evidence that individuals who received music lessons show enhanced verbal (but not visual) memory (Ho et al., 2003) and intelligence (Moreno et al., 2011, Schellenberg, 2004, Schellenberg, 2006). Further, bilinguals possess a cognitive advantage towards attenuation of irrelevant information (inhibition), self-monitoring and intelligence, providing evidence for improved general executive functions in non auditory tasks (Bialystok and Viswanathan, 2009, Festman et al., 2010, Poarch and van Hell, 2012).

Previous research on the interaction between music and language has focused on their syntactic and semantic elements, underestimating the role of pitch. The present study aims to fill this crucial gap by investigating the neural correlates (ERPs) of shared processing between melody and prosody and how they differ between musicians and nonmusicians, using a simultaneous auditory presentation paradigm. More specifically, the EEG and the reaction times of musicians and nonmusicians in a statement/question discrimination task were recorded, in order to reveal potential effects of melodic expectancy violations on prosodic processing.

Expectations for future events arise from learning the statistical properties of the auditory environment, in music (Dienes and Longuet-Higgins, 2004, Loui et al., 2010) and language (François and Schön, 2014, Maye et al., 2002), as also shown from studies demonstrating that song (merging of music and speech) facilitates language learning and stream segmentation (François et al., 2013, Francois and Schön, 2010, Schön et al., 2008). In line with the aforementioned research, in our study, we used a computational model of melodic pitch expectancy (Carrus et al., 2013, Pearce, 2005), which assumes that listeners' expectations are based on learning of statistical regularities in the musical environment. This model predicts upcoming events in an evolving melody based on the frequency with which each of these events has followed the context in a previously given corpus of music. It assumes that listeners’ expectations are based on learning of statistical regularities in the musical environment, and are predicted successfully by the computational model (Wiggins and Pearce, 2006). Thus, high-probability notes are perceived as expected and low-probability notes as unexpected (Pearce et al., 2010). The degree of expectedness of the final notes can be expressed in units of information content, which is the negative logarithm, to the base 2, of the probability of an event occurring (MacKay, 2003). The final note of low-probability melodies had a higher information content (*M*=11.75, *SD*=2.27) than the information content of high-probability melodies (*M*=1.98, *SD*=1.71).

Linguistic stimuli consisted of speech utterances differing only in their final pitch: if falling they constituted a statement, if rising a question. As suggested by Ma et al. (2011), listeners’ expectations are biased towards the perception of statements: first, because in English language statements are more frequently used than declarative questions (Bartels, 2014; Grabe, 2004), and, second, because declarative questions do not possess any word change (no inversion or wh- word) and are, thus, syntactically identical to statements (Bartels, 1997; Bartels, 2014; Grabe et al., 2003). Because it is less likely that a declarative syntax ends with interrogative intonation, we considered that statements are more expected compared to questions.

In summary, we investigated behavioural and neural responses to expectancy violations of simultaneously presented melodies and speech utterances, and how these differed between musicians and nonmusicians. Following previous literature, we hypothesised that (a) reaction times in the behavioural statement/question discrimination task will be faster when expectancy violations in language and music are presented simultaneously (an unexpected event in music might facilitate the recognition of unexpected events in speech, i.e. questions); (b) musicians’ performance will be faster and more accurate than nonmusicians', due to their increased pitch sensitivity; (c) prosodic violations will elicit the ‘prosodic expectancy positivity’ and the P600 component, reflecting prosodic reanalysis and integration processes; and (d) musicians will show enhanced early ERP components, as well as increased late positivities in response to unexpected events, reflecting enhanced pitch sensitivity due to the neuroplastic effects of musical training. Based on the existing evidence for overlapping neural resources between music and language and bidirectional influences between music and language skills in the pitch dimension, we expected that melodic expectancy would interact with language processing. Finally, following previous studies showing enhanced pitch encoding in musicians, it could be suggested that interaction effects may differ between musicians and nonmusicians.

## 2 Results

### 2.1 Behavioural findings

**Fig. 1:**
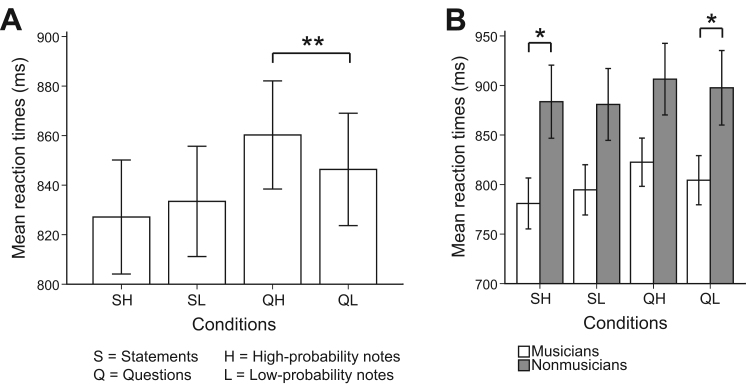
(a) Bar chart of mean reaction times (ms) for each condition (SH=statements on high-probability notes, SL=statements on low-probability notes, QH=questions on high-probability notes, and QL=questions on low-probability notes). Double asterisks (**) denote statistical significance at *p*<.01; (b) Bar chart of mean reaction times (ms) for each condition for musicians (white) and nonmusicians (gray). Error bars represent ±1 standard error mean (*SEM*).

We calculated mean reaction times for each participant across four conditions: SH (statements on high-probability notes), SL (statements on low-probability notes), QH (questions on high-probability notes), QL (questions on low-probability notes), and the results are shown in Fig. 1. A mixed factorial ANOVA on reaction times was performed (see Section 4). We found participants were significantly faster in response to statements than to questions (*prosody: F*(1,38)=24.89, *p*<.001, *η*^*2*^=.40), and musicians were overall faster than nonmusicians (*musical training*: *F*(1,38)=4.59, *p*=.039, *η*^*2*^=.11) (Fig. 1b). Further, there was a significant interaction between *prosody* and *note-probability* (*F*(1,38)=6.53, *p*=.015, *η*^*2*^=.15), and this was primarily due to the difference between questions (QH–QL: *t*(39)=2.93, *p*=.006), but not between statements (*p*>.05). Specifically, reaction times were shorter when statements were paired with a high-probability note, compared to when statements were paired with a low-probability note. The opposite effect was observed for questions, namely reaction times were longer when paired with a high-probability note than a low-probability note. Therefore, reaction times across groups were longer when expectancy in music and language was incongruent, i.e. statements were paired with low-probability notes and questions with high-probability notes.

**Fig. 2:**
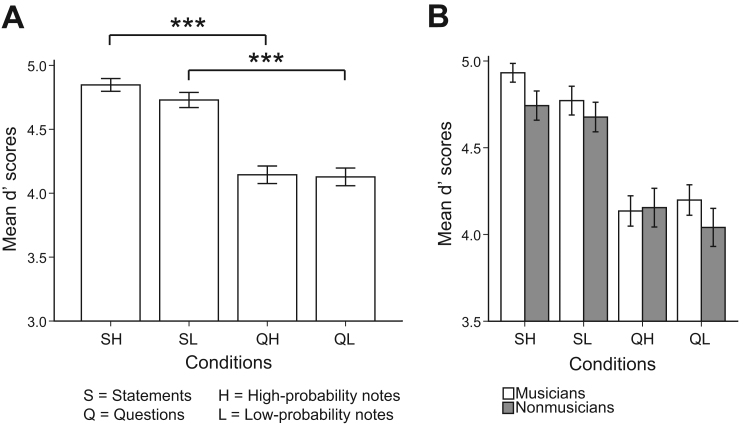
(a) Bar chart of mean *d’* scores for each condition. Triple asterisks (***) denote statistical significance at *p*<.001; (b) Bar chart of mean *d’*' scores for each condition for musicians (white) and nonmusicians (gray). Error bars represent ±1 standard error mean (*SEM*).

Mean *d'* scores for each of the four conditions are displayed in Fig. 2a. We found that the type of prosody made a significant difference between conditions (QH–SH, Wilcoxon *Z*=−5.31, *p*<.001, and QL–SL, *Z*=−4.95, *p*<.001). A marginal significance was found between SL–SH (*Z*=−1.87, *p*=.062) but no significant difference was found between QL–QH (*p*>.05). Fig.2b shows the mean accuracies for two groups. There was a marginal significance in the statements on high-probability-notes condition (SH) (Mann-Whitney *U*=−1.87, *p*=.062), but no significance in the other conditions (*p*>.05).

### 2.2 ERPs

#### 2.2.1 N1 time window (60–130 ms)

**Fig. 3:**
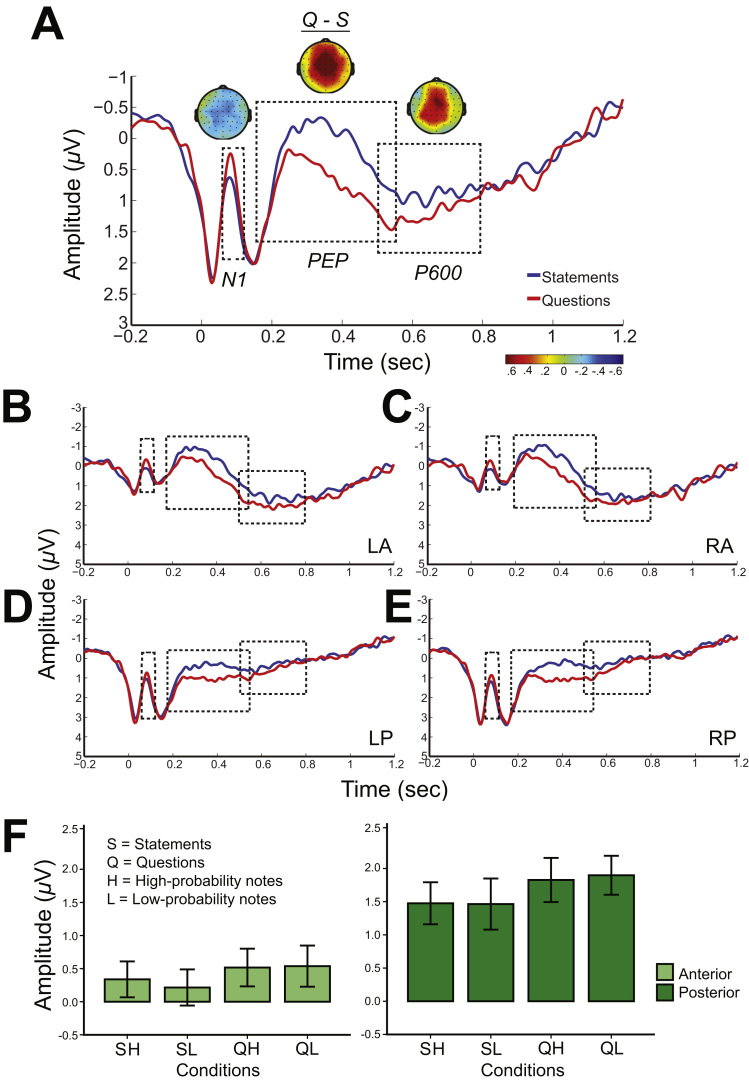
(a) Grand average ERPs (all ROIs) following statements (blue) and questions (red). The time windows with a main effect of prosody are indicated by a rectangle (from left to right: N1, prosodic expectancy positivity (‘PEP’), and P600). The difference scalp maps represent statements subtracted from questions, averaged across participants and electrodes. The grand average ERPs are displayed separately for the four ROIs in: (b) LA (left anterior), (c) RA (right anterior), (d) LP (left posterior) and (e) RP (right posterior); (f) Mean amplitudes averaged over the ROIs of anterior (light green) and posterior (dark green) electrode sites within the P600 time window in all four conditions. Error bars represent ±1 standard error mean (*SEM*). (For interpretation of the references to color in this figure legend, the reader is referred to the web version of this article.)

Within the N1 time window, ERP amplitudes were shown to be more negative in response to questions compared to statements (see Fig. 3). This was confirmed by the mixed ANOVAs, which yielded a significant main effect of *prosody* (*F*(1,37)=5.23, *p*=.028, *η*^*2*^=.12). In addition, there was a main effect of *region* (*F*(1, 37)=86.48, *p*<.000, *η*^*2*^=.70), and a marginal effect of *musical training* (*F*(1, 37)=4.00, *p*=.053, *η*^*2*^=.10). There was no effect of *note-probability* or interaction between *prosody* and *note-probability* (*F*<.29, *p*>.592). These results showed an enhanced N1 component following violation of expectations in speech prosody.

Furthermore, there was a significant *prosody*×*musical training*×*hemisphere* interaction (*F*(1, 37)=5.25, *p*=.028, *η*^*2*^=.12). In order to explore further this interaction, we carried out planned contrasts between Q–S in the left, and Q–S in the right hemisphere, for musicians and nonmusicians. The results revealed that mean amplitude difference between questions and statements was higher in the left hemisphere (*M*=−.25, *SE*=.17) (*p*>.050) than in the right hemisphere (*M*=−.13, *SE*=.14) (*p*>.050) for musicians, whereas it was lower in the left hemisphere (*M*=−.18, *SE*=.17) (*p*>.050) than in the right hemisphere (*M*=−.40, *SE*=.14) (*t*(17)=−2.82, *p*=.012) for nonmusicians. This interaction is due to the Q–S difference in the right hemisphere for nonmusicians.

#### 2.2.2 P200 time window (100–200 ms)

Musicians showed lower ERPs in comparison to nonmusicians within the P200 time window, as confirmed by the significant main effect of *musical training* (*F*(1,37)=4.34, *p*=.044, *η*^*2*^=.11). There was no effect of *prosody* or *note-probability* or interaction between these factors (*F*<1.09, *p*>.304). There was a main effect of *region* (*F*(1,37)=115.99, *p*<.001, *η*^*2*^=.76), as well as a *region*×*hemisphere* interaction (*F*(1,37)=7.59, *p*=.009, *η*^*2*^=.17). In addition, the mixed ANOVA revealed a significant interaction between *prosody*, *note-probability*, and *hemisphere* (*F*(1,37)=5.24, *p*=.028, *η*^*2*^=.12). This interaction was further analysed with planned contrasts between the left and right hemispheres in all conditions (SH left – SH right, SL left – SL right, QH left – QH right, QL left – QL right). The analysis revealed that the effect of hemisphere was important in conditions SL (*t*(38)=2.18, *p*=.035) and QH (*t*(38)=2.12, *p*=.041), where the right was significantly more positive than the left hemisphere (SL (right-left): *M*=.21, *SE*=.10, QH (right-left): *M*=.18, *SE*=.55). Conditions SH and QL did not show significant differences between the two hemispheres (*p*>.200). In summary, in the P200 time window, the right hemisphere shows significantly higher ERP amplitudes when music and language expectancy is in a different direction, i.e. language is expected (statements) and music is unexpected (low-probability notes) (SL) or the opposite (questions on high-probability notes) (QH).

#### 2.2.3 Prosodic expectancy positivity (‘PEP’) time window (150–550 ms)

**Fig. 4:**
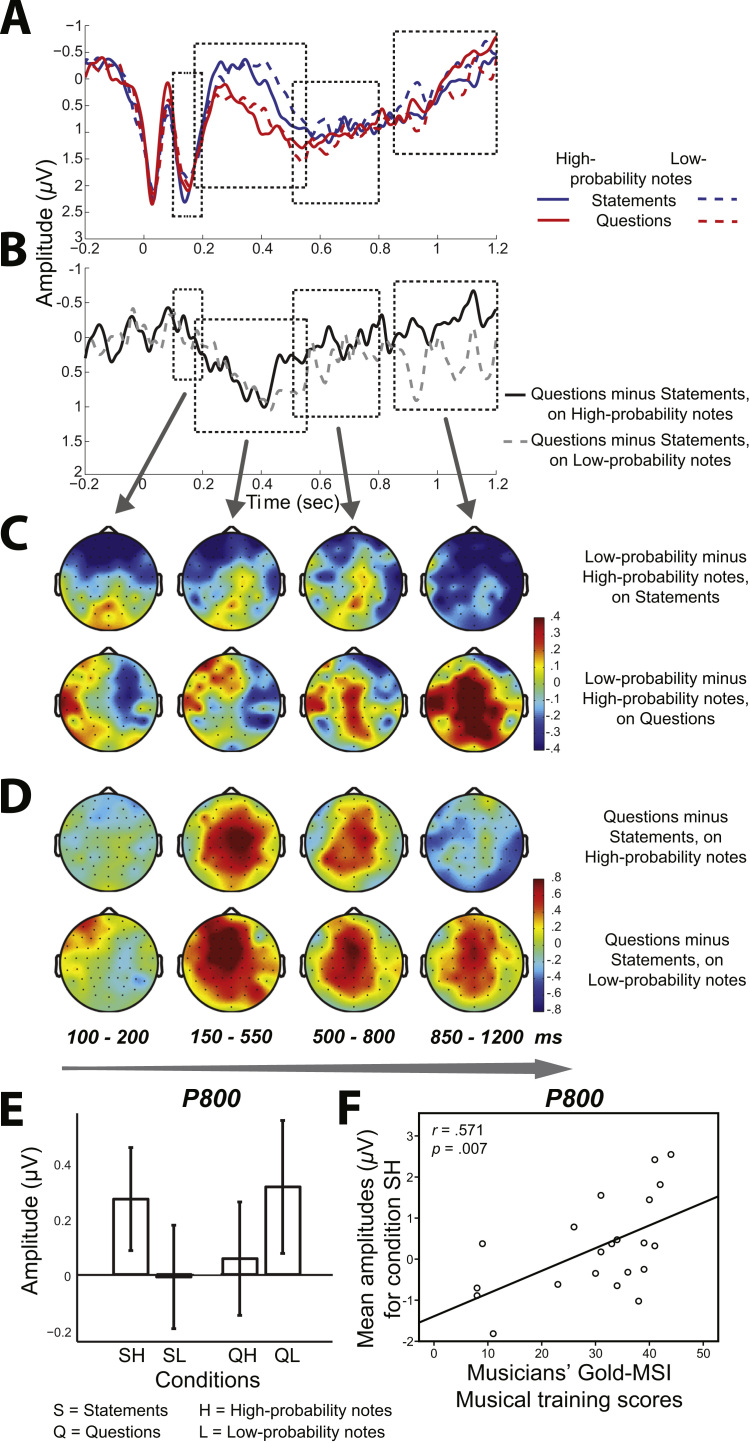
(a) Grand average ERPs (all ROIs) for all conditions: statements on high-probability notes (solid blue line), questions on high-probability notes (dotted blue line), statements on low-probability notes (solid red line), and questions on low-probability notes (dotted red line). Music-language interaction time windows are indicated with a rectangle (P200, ‘PEP’, P600, and P800); (b) Difference grand average ERPs (all ROIs) for statements subtracted from questions on high-probability notes (QH–SH) (dotted black line), and statements subtracted from questions on low-probability notes (QL–SL) (dotted gray line). The scalp topographies corresponding to the differences between conditions are presented in: (c) Low-probability minus high-probability notes on statements (SL–SH), low-probability minus high-probability notes on questions (QL–QH), and (d) questions minus statements on high-probability notes (QH–SH), questions minus statements on low-probability notes (QL – SL). These represent averages across participants and electrodes; (e) Mean amplitudes averaged over ROIs within the P800 time window in all four conditions. Error bars represent ±1 *SEM*; (f) Correlation between musicians' amount of musical training (Gold-MSI Musical Training score) and their mean ERP amplitudes (condition SH) within the P800 time window. (For interpretation of the references to color in this figure legend, the reader is referred to the web version of this article.)

As compared with statements, a larger ERP positivity between 150–550 ms, with fronto-central scalp distribution was observed for questions (see Fig. 4). The mixed ANOVA revealed a significant main effect of *prosody* (*F*(1,37)=19.65, *p*<.001, *η*^*2*^=.35) and *region* (*F*(1,37)=25.07, *p*<.001, *η*^*2*^=.40). Questions showed an enhanced PEP (*M*=1.27, *SE*=.25) in the anterior ROIs (LA, RA) compared to statements (*M*=.75, *SE=*.22), as well as in the posterior ROIs (LP, RP) (Q: *M*=.40, *SE*=.20, S: *M*=−.48, *SE*=.16). There was no effect of *note-probability* or *musical training* (*F*<.26, *p*>.616). The results confirmed the presence of an increased positivity in response to questions compared to statements in the PEP time window, which was more enhanced in the anterior sites.

Furthermore, the ANOVA revealed a significant interaction involving *prosody*, *note-probability* and *hemisphere* (*F*(1,37)=4.67, *p=*.037, *η*^*2*^=.011) (Fig. 4d). Further analyses with planned contrasts between conditions QH–SH, and QL–SL, on the left and right hemisphere, revealed that mean amplitude difference between questions and statements was significantly higher on low-probability notes (*M*=.66, *SE*=.20) (*t*(38)=3.25, *p*=.002) than on high-probability notes (*M*=.39, *SE*=1.05) (*t*(38)=2.32, *p*=.026) at the left hemisphere, whereas this difference had the opposite direction (QL–SL: *M*=.51, *SE*=.20, (*t*(38)=2.61, *p*=.013), QH–SH: *M*=.58, *SE*=.17, (*t*(38)=3.50, *p*=.001)) at the right hemisphere. In summary, low-probability notes in the left hemisphere elicit an enhanced PEP component compared to high-probability notes, whereas the opposite is true for the right hemisphere (high-probability notes are more positive than low-probability notes).

Finally, a significant interaction between *prosody*, *musical training* and *hemisphere* (*F*(1,37)=8.24, *p*=.007, *η*^*2*^=.18) was found, which was further analysed with planned contrasts between Q–S in the left, and Q–S in the right hemisphere, for musicians and nonmusicians. The results revealed that mean amplitude difference between questions and statements was higher in the right hemisphere (*M*=.51, *SE*=.18) (*t*(20)=2.90, *p*=.009) than in the left hemisphere (*M*=.28, *SE*=.16) (*p*=.087, marg.) for musicians, whereas it was lower in the right hemisphere (*M*=.58, *SE*=.19) (*t*(17)=3.06, *p*=.007) than in the left hemisphere (*M*=.81, *SE*=.21) (*t*(17)=3.80, *p*=.001) for nonmusicians. In summary, for musicians the Q–S amplitude difference was higher in the right hemisphere, whereas for nonmusicians it was higher in the left hemisphere.

#### 2.2.4 P600 time window (500–800 ms)

As with the PEP time window, ERP amplitudes in response to questions were shown to be more positive compared to statements, from 500 to 800 ms after the onset of the critical word/note (see Fig. 3). An ANOVA in this time window revealed significant main effects of both *prosody* (*F*(1,37)=7.07, *p*=.012, *η*^*2*^=.16) and *region* (*F*(1,37)=38.49, *p*<.001, *η*^*2*^=.51), but no effect of *note-probability* or *musical training* (*F*<1.76, *p*>.193). As shown in Fig. 3f, positivity was enhanced in questions, and was also greater in the posterior ROIs.

**Fig. 5:**
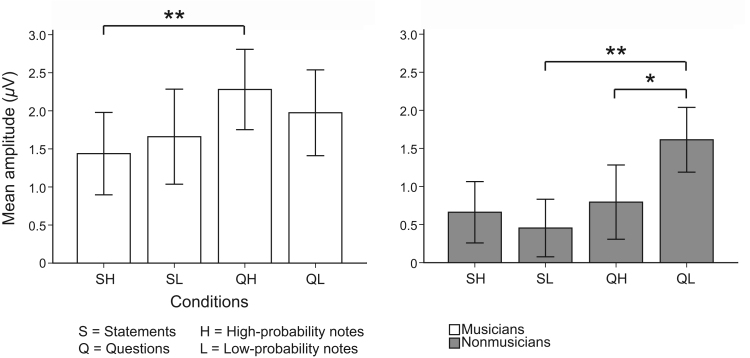
Error bars with the mean ERP amplitude values for all conditions in the P600 time window (500–800 ms) (from left to right: statements on high-probability notes (SH), statements on low-probability notes (SL), questions on high-probability notes (QH), and questions on low-probability notes (QL)), for musicians (white) and nonmusicians (gray). Error bars represent ±1 *SEM*.

Furthermore, the analysis revealed a significant interaction between *prosody*, *note-probability* and *musical training* (*F*(1,37)=5.62, *p*=.023, *η*^*2*^=.13). As shown from the difference topoplots for the P600 time window (Fig. 4b and c), the *prosody*×*note-probability* interaction is mainly focused at mid-frontal sites. In order to compare differences between musicians and nonmusicians within this time window, we run a further 2 (*prosody*: statement vs. question)×2 (*note-probability*: high-probability vs. low-probability) ×2 (*musical training*: musicians vs. nonmusicians) mixed ANOVA at the peak electrodes of the interaction (Fz, FCz, Cz, CPz, C1, C2) (see Fig. 5). Questions elicited significantly more positive responses compared to statements (main effect of *prosody*: *F*(37)=14.93, *p*<.001, *η*^*2*^=.29). As expected, there was a significant interaction between *prosody*, *note-probability* and *musical training* (*F*(37)=5.21, *p*=.028, *η*^*2*^=.12). In order to explore further this interaction, planned contrasts were carried out between conditions SH–SL, QH–QL, SH–QH and SL–QL, for musicians and nonmusicians. As shown in Fig. 5, in musicians the interaction was due to the difference between high-probability melodies (QH–SH: *t*(20)=3.17, *p*=.005), whereas in nonmusicians the difference was important between low-probability melodies (QL–SL:*t*(17)=1.16, *p*=.004), as well as questions (QL–QH: *t*(17)=.82, *p*=.031).

#### 2.2.5 P800 time window (850–1200 ms)

A significant *note-probability*×*musical training* interaction was observed (*F*(1,37)=4.61, *p*=.039, *η*^*2*^=.11), which was further analysed with planned contrasts between high- and low-probability notes for the two groups. The results showed that mean ERP amplitudes for musicians were higher in response to high- compared to low-probability notes, whereas the opposite effect was observed for nonmusicians. However, neither of the contrasts was significant (*p*>.535). The ANOVA also revealed a significant interaction between *prosody* and *note-probability* (*F*(1,37)=5.91, *p*=.02, *η*^*2*^=.14) (Fig. 4e). In order to explore further this interaction, planned contrasts were carried out between conditions SH–SL, QH–QL, SH–QH and SL–QL. Note-probability was found to be important only in statements (SH–SL: *t*(38)=1.98, *p*=.055), but not in questions (QH–QL: *p*>.05). Prosody made a marginally significant difference in the unexpected conditions (SL–QL: *t*(38)=−1.80, *p*=.080), but not in the expected conditions (*p*>.05). The time course of music and language interactions in the consecutive time windows mentioned above is illustrated in Fig. 4. There was no effect of *prosody* or *note-probability* (*F*<.29, *p*>.594). A main effect of *region* was found (*F*(37)=40.00, *p*<.001, *η*^*2*^=.52).

Furthermore, we observed large variability in the P800 amplitudes across participants (as shown from the error bars in Fig. 4e). In order to further investigate this observation, we run a Pearson product-moment correlation between participants’ (musicians and nonmusicians) Gold-MSI Musical Training scores and their mean ERP amplitudes. Interestingly, significant correlations were found between the ERP amplitudes of musicians and their level of musical training (SH: *r*=.571, *p*=.007; SL: *r*=.525, *p*=.015; QH: *r*=.377, *p*=.092 (marginal); QL: *r*=.527, *p*=.014): the higher the level of musical training, the more positive was the ERP response. In contrary, no significant correlations were found between nonmusicians’ Gold-MSI scores and their ERP amplitudes (*r*<.200 and *r*>−.200, *p*>.05). The strongest positive correlation between musicians’ amount of musical training and their ERP amplitude was observed for condition SH, and is illustrated in Fig. 4f.

#### 2.2.6 ERPs and reaction times association

Potential associations between ERP components and reaction times (RTs) in the statement/question discrimination task were subsequently examined. More specifically, we wanted to investigate whether any specific ERP component could successfully predict faster RTs when questions were paired with low-probability notes (QL) than when questions were paired with high-probability notes (QH) (QH–QL: *t*(39)=2.93, *p=*.006). We assumed that an ERP component which predicts RTs should precede in time the lowest RT observed (i.e. ERPs occurring after an RT cannot be used to predict it). The fastest RT across participants in the QH and QL conditions was 608.53 ms. Therefore, we examined potential associations between RTs and the ERPs preceding this minimum RT: N1 (60–130 ms), P200 (100–200 ms) and PEP (150–550 ms).

**Fig. 6:**
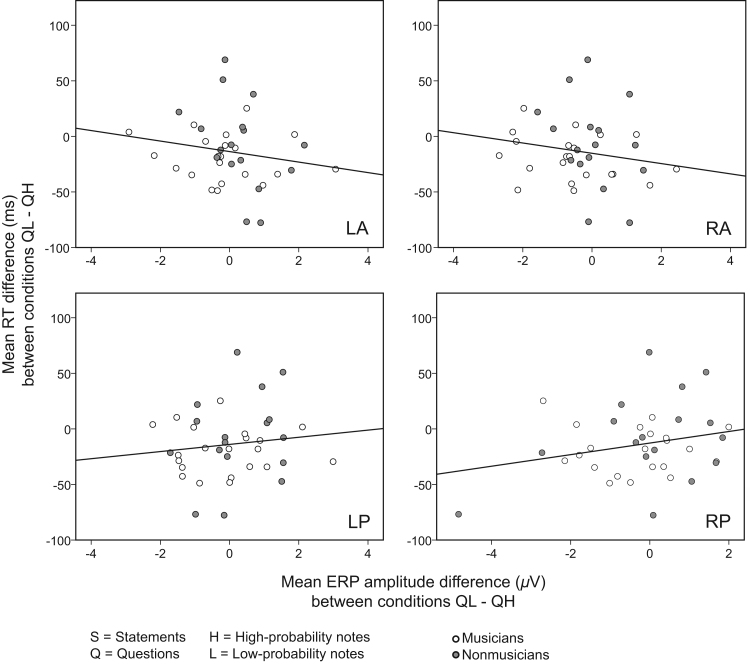
Correlations between mean ERP amplitude differences (*μ*V) between conditions QL (questions on low-probability notes) and QH (questions on high-probability notes), and mean RTs (reaction times) (ms) between the same conditions in the behavioural task, for musicians (white) and nonmusicians (gray). The scatterplots correspond to the four ROIs (LA=left anterior, LP=left posterior, RA=right anterior, RP=right posterior).

Linear backward regression was performed to predict RTs from each of the three ERP components mentioned above. The mean amplitude difference between conditions QL and QH across participants in each of the four ROIs (LA, LP, RA, RP) was the predictor variable. The mean difference of RTs between conditions QL and QH across participants was the dependent variable. Neither N1 nor PEP time-window QL–QH amplitudes significantly predicted the difference in RTs (*p*>.220). Interestingly, regression models using the P200 significantly predicted RTs. Specifically, a model with RA, RP and LA ROIs as predictors significantly predicted RTs (*R*^*2*^=.21, *F*(3,34)=3.02, *p*=.043). Only the RP ROI was found to be significant for the prediction (*p*=.009). Another model including only the right ROIs (RA, RP) predicted more significantly the dependent variable (*R*^*2*^=.20, *F*(2,35)=4.47, *p*=.019). Here both RA and RP ROIs added significantly to the prediction (*p=*.017, and *p*=.009, respectively). All four ROIs as predictors showed a marginally significant model (*R*^*2*^=.22, *F*(4,33)=2.30, *p*=.080) (Fig. 6), in which none of the four ROIs was statistically significant (*p* > .216).

## 3 Discussion

The present study investigated the interactions between melodic expectancies and prosodic expectancies (statements, questions) using a statement/question discrimination task in musicians and nonmusicians, and demonstrated their behavioural and neural correlates. Behavioural results showed that musicians had superior performance to nonmusicians, providing evidence for transfer of pitch processing abilities from the music to the speech domain. At the neural level, questions were associated with increased N1 negativity compared to statements, as well as with a late fronto-central positivity (prosodic expectancy positivity), reflecting prosodic re-analysis processes. Furthermore, when violations occurred in both music and language (double violation: questions on low-probability notes), a linear regression model significantly predicted faster reaction times from the corresponding ERPs within the P200 time window. Importantly, musicians showed lower P600 in the double violation condition, suggesting the usage of fewer neural resources to process strong pitch incongruities. Critically, musicians’ P800 amplitudes were proportional to their level of musical training, suggesting that this expertise might shape the pitch processing of language. We speculate that the latter beneficial aspect of musical training could be explained by its intrinsic strengthening effect on general executive functions (e.g., attention and working memory).

In this section, we will first examine the effect of prosody on task performance and on neural responses to statements and questions. Then, we will focus on the interactions between prosody and note-probability, and finally, the role of musical expertise on pitch processing and cognition will be discussed.

### 3.1 Effect of prosody

Overall, statements were recognised faster and more accurately across groups, reflecting a bias towards expected prosody. Similar findings, in a statement/question discrimination task, have been previously reported in different languages (Ma et al., 2011, Peters and Pfitzinger, 2008). Moreover, higher recognition accuracy towards grammatically (Colé and Segui, 1994) or syntactically correct sentences (Hoch et al., 2011, Slevc et al., 2009) has been previously associated with the unexpectedness of the incorrect sentences. Similarly, higher accuracy has been observed for semantically neutral sentences as opposed to sentences with a violation of emotional or linguistic prosody (Paulmann et al., 2012). Noteworthy, in the aforementioned studies, expectancy was expressed by correct/incorrect sentences, whereas in our study for the same purpose we used statements/questions. This methodological difference might underlie not exactly comparable levels of expectancy, as incorrect sentences might be perceived as more unexpected than questions; however we assumed that our methodological choice would allow us to study the effect of language expectancy in a categorical fashion (expected vs. unexpected).

Although we did not have any specific hypothesis about early ERP responses, the results showed increased negativity within the N1 component in response to questions, which is in line with previous findings on unexpected events (Debener et al., 2005, Horváth et al., 2008, Proverbio et al., 2004). As predicted, questions elicited a larger positivity at around 150–550 ms after the onset of the last word/note with a fronto-central scalp distribution. A similar ERP has been associated with violations of prosodic expectancy (prosodic expectancy positivity: PEP) (Kotz and Paulmann, 2007, Paulmann et al., 2012). Previous contradictory findings on the lateralisation of the PEP component (Chen et al., 2011, Paulmann and Kotz, 2008), could be attributed to the different type of prosodic manipulation (linguistic or emotional), as well as to the different task demands (Wildgruber et al., 2004). With regards to the essence of the PEP deflection, we argue that it is associated with re-analysis processes of prosodically unexpected utterances (Astésano et al., 2004, Paulmann et al., 2012, Paulmann and Kotz, 2008).

### 3.2 Interactions between prosody and note-probability

As predicted, reaction times across groups were shorter when expectancy violations in music and language occurred simultaneously. Specifically, responses to statements were faster on high- than low-probability notes, and the opposite effect was observed for questions. This interaction might be due to facilitation effects between music and language processing of expectation. Expected musical chords (tonics) have been previously found to facilitate processing of concurrent language stimuli (phonemes), which constitutes the ‘tonal function effect’ (Bigand et al., 2001, Escoffier and Tillmann, 2008). Although the present study did not involve the use of harmony, the similar findings could be attributed to the fact that melodies can also engage harmonic processing by implying an underlying chord sequence (Giordano, 2011, Koelsch, 2012).

The size of the final interval in our melodic stimuli might constitute a limitation, as the majority of the high-probability melodies had small intervals, whereas low-probability melodies had an equal amount of small and large intervals (see Section 4.2). For example, small intervals are more frequent than large intervals in Western tonal music (Krumhansl, 1995, Vos and Pasveer, 2002, Vos and Troost, 1989). Therefore, small intervals might constitute a feature *sine qua non* melodies are usually built, thus constituting an intrinsic characteristic of high-probability melodies. Faster reaction times in response to high-probability intervals may thus be related to the high frequency of small intervals. However, besides the actual pitch difference, perceived interval size has been found to depend on the level of musical training, the melodic direction, as well as whether the interval is larger than an octave (Russo and Thompson, 2005).

The faster reaction times in the double violation condition (questions on low-probability notes) were successfully predicted from the corresponding P200 component in the right ROIs, as shown from a linear regression model we implemented. Therefore, we suggest that the P200 might facilitate pitch expectancy processing in music and language. Future research could assess this hypothesis in a similar simultaneous presentation paradigm, investigating violation of grammar or syntax, instead of linguistic prosody.

Our main hypothesis was related to the investigation of the neural effect of melodic violations on prosodic processing. Interestingly, we observed neural interactions in consecutive time windows, from the P200 until the P800, suggesting interdependencies at an early stage (linked to sensory and perceptual processing (Hillyard et al., 1973; Näätänen et al., 1978)), as well as at a later stage (integration and re-analysis processes (Eckstein and Friederici, 2005)). The P800 showed the largest positivity when music and language expectancy were in the same direction, i.e. both expected (statements on high-probability notes) and both unexpected (questions on low-probability notes). The latter condition (double violation) showed the highest amplitude overall (not significant), which could been linked to fundamental frequency re-analysis processes of unexpected intonation (Astésano et al., 2004). Although Astésano et al. (2004) linked the P800 to prosodic manipulations, considering the music-language interaction observed, we argue for its amodal rather than language-specific role in detecting expectancy violations.

Previous studies have interpreted music-language interactions as a competition of neural resources used for the simultaneous processing of their syntactic elements (Carrus et al., 2013, Koelsch and Gunter, 2005). Therefore, interactions in the pitch dimension in a simultaneous presentation paradigm would suggest that melodic and prosodic expectancy are interdependent. Our study is the first to find this effect, favouring the possibility that music and language share access to a limited pool of resources for pitch processing. In particular, recent research suggested that these shared resources between music and language for pitch processing are attributed to more general higher-order cognitive mechanisms (e.g., working memory (WM)), rather than specialised lower-level pitch perception processes (Marie et al., 2011, Moreno and Bidelman, 2014, Smayda et al., 2015). In support of this hypothesis, fundamental structures of WM, such as Broca's area, premotor cortex, pre-SMA/SMA, left insular cortex and inferior parietal lobe, are involved in both tonal and verbal WM (Schulze et al., 2011). Accordingly, in an fMRI study Janata et al. (2002) have shown that attentive listening to music recruits neural circuits serving general functions, such as attention, WM, and semantic processing. Strong evidence favouring shared higher-order, executive functions have reported enhanced attention and WM associated with musical expertise, suggesting that this strengthening of executive functions is responsible for the speech processing benefits (Besson et al., 2011, Bialystok and Depape, 2009, Carey et al., 2015, George and Coch, 2011, Parbery-Clark et al., 2012, Patel, 2014, Rigoulot et al., 2015, Smayda et al., 2015, Strait et al., 2010, Strait and Parbery-Clark, 2013). In the next Subsection 3.4 we will discuss the effects of musical training on melodic and speech pitch processing considering the potential role of general functions.

### 3.3 Effects of musical training

Confirming our hypothesis on musical expertise, musicians showed overall better performance in the statement/question discrimination task. They were significantly faster and showed a trend for higher accuracy compared to nonmusicians, which is in line with studies on increased pitch sensitivity for musicians in linguistic tasks (Alexander et al., 2005, Deguchi et al., 2012, Maess et al., 2001, Magne et al., 2006, Schön et al., 2004). For example, it has been shown that musicians are more accurate in detecting pitch violations not only in music, but also in language (Magne et al., 2003), and show better discrimination abilities between weak incongruities and congruities in both domains (Besson and Faïta, 1995, Magne et al., 2006, Schön et al., 2004). It is therefore likely that musicians are able to transfer their musical pitch processing abilities to speech pitch tasks, due to common underlying pitch processing mechanisms.

At the neural level, musicians showed overall lower P200 amplitude (linked to attention processes (Shahin et al., 2003)) compared to nonmusicians. Although this contradicts previous findings on training effects reporting enhanced P200 in musicians (Atienza et al., 2002, Tremblay et al., 2001), evidence from other cognitive domains demonstrated lower amplitude in the early ERP components explained as less attentional effort needed (Berry et al., 2010, Berry et al., 2009). That is, musicians might require reduced attentional demands (lower P200) to out-perform nonmusicians in the behavioural task (higher reaction times and accuracy), suggesting greater efficiency at prosodic pitch processing. Another possible explanation of this effect could be related to the allocation of attention, as participants were asked to focus on the speech and ignore the music. Future research could investigate this hypothesis by instructing the participants to rate the melodic endings while ignoring the language.

Importantly, we observed a neural interaction between prosodic and melodic violation and musical training, in the P600 time window. Specifically, musicians showed an overall larger positivity compared to nonmusicians. This is in line with previous literature related to harmonic violations (Besson and Faïta, 1995, Featherstone et al., 2013, Regnault et al., 2001), and prosodic or melodic violations (Schön et al., 2002). For example, Regnault et al. (2001) found enhanced late positivities elicited by dissonant chords in musicians compared to nonmusicians. Critically, we found that strong incongruities (questions on low-probability notes) elicited smaller P600 than weaker incongruities (questions on high-probability notes) in musicians, whereas nonmusicians showed the opposite pattern (non significant). This trend confirms previous studies which demonstrated that strong music and language incongruities elicit lower P600 amplitudes after auditory pitch training in musicians, but not in nonmusicians (Besson et al., 2007, Moreno and Besson, 2006). Considering that P600 reflects working memory demands (Gibson, 1998), we suggest that musicians need less neural resources to process and integrate strong pitch incongruities (Featherstone et al., 2013, Tatsuno and Sakai, 2005). In contrary, nonmusicians find simultaneous violations of expectations more demanding and difficult to integrate, due to lower working memory capacity. Therefore, we speculate that pitch processing might become automatic in musically trained people.

Further analysis revealed that Gold-MSI musical training scores of musicians (but not of nonmusicians) correlated positively with their P800 amplitudes: the higher the level of musical training, the more positive was the ERP response. This finding might provide evidence for neuroplastic changes in the pitch domain due to musical expertise (Moreno and Bidelman, 2014, Pantev et al., 2001, Ridding et al., 2000, Schlaug et al., 1995, Steele et al., 2013, Wan and Schlaug, 2010).

To sum up, we propose that expertise-related effects might result in lower-level perceptual benefits, as well as higher-order cognitive enhancement. In particular, one possibility is that musical training enhances pitch perception, and such improvement may be mediated by tuning of neurons in auditory cortical regions (Schneider et al., 2002) and the brainstem (Bidelman et al., 2011, Wong et al., 2007). Another possible explanation is that expertise results in more efficient suppression of task-irrelevant auditory stimuli. Thus, musicians could better inhibit the musical stimuli while focusing on the speech (as they were instructed), which had an impact on their allocation of attention (lower P200). This is in line with evidence about musicians demonstrating benefits in sound segregation, such as speech-in-noise conditions (Parbery-Clark et al., 2009) and the “cocktail party problem” (Swaminathan et al., 2015, Zendel and Alain, 2009). Therefore, successful inhibition of task-irrelevant stimuli might constitute one of the mechanisms of improved cognitive control following expertise.

### 3.4 Conclusion

Our findings suggest that melodic expectancy influences the processing of language pitch expectancy. We reveal that musical expertise modulates the nature of such influence, by facilitating the processing of unexpected events, and by providing a more refined response to pitch not only in music, but also in language. Critically, musicians’ neural responses were found to be proportional to their level of musical expertise, suggesting that expertise shapes prosodic processing. Therefore, these results provide evidence for the beneficial effects of musical training on general cognitive functions (e.g., allocation of attention, working memory), during the simultaneous processing of expectancy violations in music and language. We suggest that these findings have implications for investigating potential shared higher-order mechanisms between music and language.

## 4 Methods and materials

### 4.1 Participants

Forty-two neurologically healthy adult human volunteers (25 female) aged between 18 and 37 years old with mean±s.d. age of 23.79±4.25 participated in a behavioural and an EEG experiment. All participants were native speakers of English (L1), not tested for potential second language proficiency, with normal hearing and normal or corrected-to-normal vision (self-reported). Participants were divided into two groups according to their self-reported level of musical training: musicians (22 subjects, mean age of 22.59 years, 15 female) which had a mean±s.d. Gold-MSI score of 35.91±6.80, and nonmusicians (20 subjects, mean age of 24.89 years, 10 female) which had a mean±s.d. Gold-MSI score of 17.80±10.12. The ‘Goldsmith's Musical Sophistication Index’ (Gold-MSI) questionnaire was administered to validate participants' self-reported musicality (Müllensiefen et al., 2014). Participants gave written informed consent in accordance with procedures approved by the local ethics committee of the Department of Psychology at Goldsmiths, and received a monetary compensation for their participation.

### 4.2 Materials

The Gold-MSI assesses musical engagement and behaviour. For our group validation, we used the ‘Musical Training’ factor, the Gold-MSI Dimension 3, which includes seven statements regarding formal musical training experience and musical skill, and has a possible score of 7–49 points. Each statement (e.g., ‘I have never been complimented for my talents as a musical performer’) requires a response on a 7-point scale ranging from 1 (*Completely Disagree*) to 7 (*Complete Agree*).

**Fig. 7:**
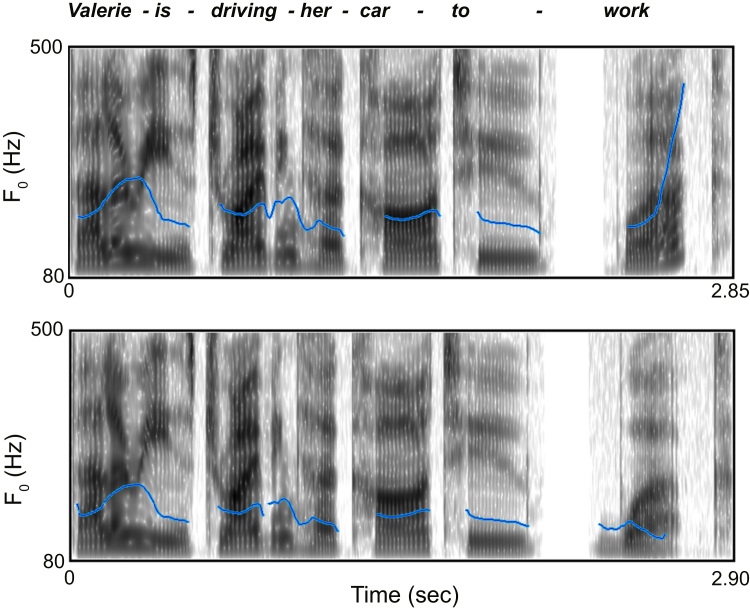
Two paired stimuli illustrating the different intonation conditions in the experiment. These are the voice spectrograms with their fundamental frequency contours (blue line). Top: the fundamental frequency *F*_*0*_ (Hz) of the final word rises, thus forming a question (*Valerie is driving her car to work?*); bottom: the fundamental frequency *F*_*0*_ (Hz) of the final word falls, thus forming a statement (*Valerie is driving her car to work.*). Figures were created using the PRAAT software (Boersma, 2001). (For interpretation of the references to color in this figure legend, the reader is referred to the web version of this article.)

Linguistic stimuli consisted of 200 seven-word utterances equally divided into two conditions: (i) statements, if the last word had a falling pitch, and (ii) questions, if it had a rising pitch. Therefore, the two conditions differed only in the final pitch (see Fig. 7 for an illustration of the intonation contours produced by the statement and question version of a typical utterance). One prominent global rule governing statement/question discrimination is that listeners tend to perceive utterances with a final pitch rise as questions, whereas utterances with a final fall as statements (Studdert-Kennedy and Hadding, 1973). Questions that are syntactically declarative sentences are commonly used (besides wh- and yes/no questions) in different English dialects, as well as American English (Bartels, 2014; Grabe et al., 2003; Grabe, 2004). The absence of word-order change in declarative questions results in statement/question discrimination judgments based mainly on the final pitch contour, which is typically rising (Bartels, 1997; Bartels, 2014). As suggested by Ma et al. (2011), listeners’ expectations are biased towards the perception of statements: first, because in English language statements are more frequently used than declarative questions (Bartels, 2014; Grabe, 2004), and, second, because declarative questions do not possess any word change (no inversion or wh- word) and are, thus, syntactically identical to statements (Bartels, 1997; Bartels, 2014; Grabe et al., 2003). Because it is less likely that a declarative syntax ends with interrogative intonation, we therefore considered that statements are more expected compared to questions.

The language stimuli were recorded by a female opera singer experienced in musical theatre and also a native speaker of British English. She was asked to pronounce the utterances in a natural way. The recording took place in a soundproof booth and a Zoom H4n recorder was used for this purpose (mono, 44.1 kHz, 24-bit recording). The 200 spoken sentences were recorded in pair, so that they were lexically identical but differed in prosody. Using WaveLab software, sentences were normalised to the same amplitude (the lower-amplitude sentence of a statement-question pair was scaled up in amplitude to match the higher). In order to control for differences between statements and questions other than the pitch of the final word, half of the statements were considered ‘stems’ for the paired questions, the last words of which were attached to them. Half of the questions were ‘stems’ for their paired statements (following the method of Patel et al., 1998a). PRAAT software was used for this purpose.

Musical stimuli consisted of 200 five-note isochronous melodies ending either with a high-probability or a low-probability note (Carrus et al., 2013), created using Pearce's (2005) computational model of melodic expectation. Specifically, the model created low-probability final notes for high-probability melodies, by choosing a new pitch with a probability lower than that of the actual continuation. Half of the final notes of the low-probability melodies produced were preceded by large intervals of six or more semitones, whereas the rest were preceded by small intervals of less than six semitones (Carrus et al., 2013). In order to investigate potential differences between note-probability (high vs. low) and interval size (small vs. large), we performed a chi-square test which revealed a significant relationship between the two variables (*χ*^*2*^(1)=55.64, *p*<.001). Specifically, only 7% of the high-probability melodies had large final intervals, in contrast to low-probability melodies of which 56% had large intervals. This intrinsic characteristic of high-probability melodies to use small final intervals is confirmed by studies revealing that the majority of closures of melodic sequences in Western tonal music consist of one or two semitones (Krumhansl, 1995, Vos and Pasveer, 2002).

**Fig. 8:**
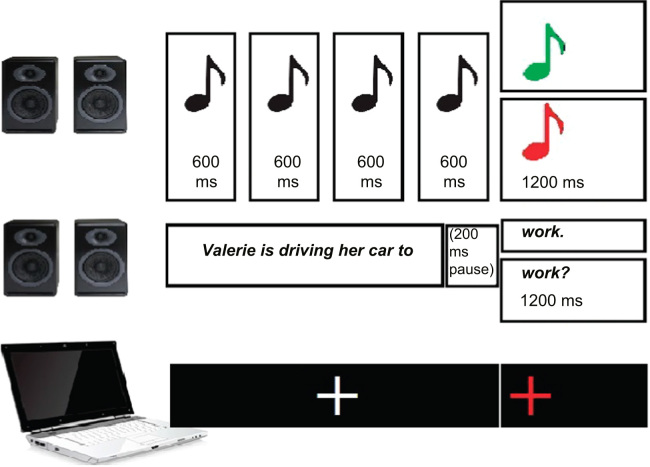
An illustration of the experimental design. Speech and melodies are presented simultaneously via speakers. Participants listened simultaneously to seven-word sentences and five-note melodies. The linguistic stimuli ended with a falling pitch (statements) or with a rising pitch (questions). Melodies ended with either a high- or a low-probability note. The onset of the final word was the event of interest in the analysis. A fixation cross in the centre of the screen was shown during stimuli presentation, and turned red at the onset of the last word/note.

The linguistic and melodic stimuli were played binaurally, both left and right, and had the same volume (Fig. 8). The presentation time was 600 ms for each of the first four musical notes and 1200 ms for the final note. As the temporal distribution of syllables is not isochronous in speech, an isochronous presentation of the sentences' words would result in utterances sounding unnatural (Geiser et al., 2008). Care was taken so that the onset of the final note coincided with the onset of the utterances' final word by inserting a 200 ms pause between the penultimate and the final word. Overall, there were 400 trials, as each of the 200 linguistic stimuli was presented twice: once combined with the 100 high-probability melodies and once with the 100 low-probability melodies. The pairing of a specific linguistic stimulus with a melodic stimulus, as well as the presentation order, was randomised across participants. During the simultaneous presentation of the auditory language and melodic stimuli, a fixation cross was presented at the centre of the screen. Two speakers (Creative Gigaworks, Creative Technology Ltd.) were used for the stimuli reproduction, and the volume was kept constant across participants and for the duration of the experiment. Cogent 2000 (www.vislab.ucl.ac.uk/cogent.php), a MATLAB (MATLAB 7.12.0, The MathWorks, Inc., Natick, MA, 2011) Toolbox was used to present the stimuli.

### 4.3 Procedure

All participants took part in two separate experiments: one EEG and one behavioural experiment. Participants completed first the EEG and then the behavioural task in order to avoid neural habituation to the stimuli; a 15-min break was provided between the two sets of experiments.

In order to investigate whether participants' neural responses changed depending on the type of melody (expected or unexpected) the utterances were paired with, their EEG was recorded. At the beginning all participants completed the Gold-MSI questionnaire. Then they were seated in front of a computer in a dimly lit room. Through written instructions, they were informed that they would listen simultaneously to speech and melodies. They were instructed to attend only to the speech, ignoring the music. They were informed about the different sentence types, but not about the different melody types. For the EEG experiment, they were prompted only for 10% of trials to indicate whether the spoken sentence they heard was a statement or a question by pressing two buttons of a response box. The inter-trial interval was randomised between 1.5 and 2 s. Two practice trials (one statement, and one question) familiarised them with the task. Breaks were provided after each of the four blocks of 100 trials (about 12 min). Across participants the presentation order of the trials was randomised, and each sentence was randomly paired with a melody. At the end of the EEG, participants performed in the behavioural version of the experiment in which almost identical procedures were followed, except participants had to indicate at every trial as fast and accurate as possible the type of sentence (statement or question), by pressing two keys in the keyboard. The overall procedure lasted for approximately 2 h.

### 4.4 EEG recording and pre-processing

The EEG signals were recorded with sixty-four Ag-AgCl electrodes placed according to the extended 10–20 electrode system (Jasper, 1958) and amplified by a BioSemi ActiveTwo amplifier (www.biosemi.com). The vertical and horizontal EOGs were recorded in bipolar fashion, in order to monitor eye-blinks and horizontal eye-movements. The EEG recording used a sampling frequency of 512 Hz, and the signals were band-pass filtered between .16 and 100 Hz. MATLAB Toolbox EEGLAB (Delorme and Makeig, 2004) was used for data preprocessing, and FieldTrip (Oostenveld et al., 2011) for data analysis. EEG data were re-referenced to the algebraic mean of the right and left earlobe electrodes (Essl and Rappelsberger, 1998). Continuous data were high-pass filtered at .5 Hz and then epoched from −1000 ms to 2000 ms time-locked to the onset of the last word/note. Artefact rejection was done in a semi-automatic fashion. Specifically, independent component analysis was run to correct for eye-blink related artefacts. Data from electrodes with consistently poor signal quality, as observed by visual inspection and by studying the topographical maps of their power spectra, was then removed and reconstructed by interpolation from neighbouring electrodes (5.65% of the data). Subsequently, epochs containing amplitude exceeding ±85 *μ*V were removed after visual inspection. Three participants were removed due to poor EEG data quality (more than 25% of the trials rejected). Additional preprocessing included low-pass filtering the epoched data at 30 Hz, and baseline correcting to 200 ms prior to last word/note onset.

### 4.5 Statistical analysis

*Behavioural data:* As the percentage of correct responses would constitute a biased measure of accuracy, signal detection theory was used to score discriminability (d prime scores, or *d*') at the statement/question task (Macmillan and Creelman, 2005). Hits (correctly recognised statements) and false alarms (falsely recognised questions) were calculated for each experimental condition across participants (statements on high-probability notes (‘SH’), statements on low-probability notes (‘SL’), questions on high-probability notes (‘QH’), and questions on low-probability notes (‘QL’)). All 100% and 0% scores were altered to 99.50% and .50%, in order to correct for ceiling and floor effects, respectively. Mean reaction times and d prime (*d*') accuracy scores were calculated for each condition across participants. Reaction times were analysed for correct trials only; trials with reaction times above and below two standard deviations were considered as outliers and removed from subsequent analysis (Ratcliff, 1993). First, a 2x2×2 mixed ANOVA was performed with *prosody* (statement, question) and *note-probability* (high, low) as within-subjects factors, and *musical training* (musicians, nonmusicians) as the between-subjects factor. In order to further explore the prosody×note-probability interaction, planned contrasts were run between conditions. As the accuracy scores were non-normally distributed (*p*<.05, Shapiro-Wilk test), non-parametric test (Wilcoxon signed-rank) test was used for planned contrasts in *d*' scores. All follow-up tests were Bonferroni corrected. Two outliers were identified by inspection of boxplots. One-sample *t* tests confirmed that the accuracy of these two participants was significantly different compared to the accuracy of the rest of the participants in the QE condition (*t*(39)=22.02, *p*<.001) and the QU condition (*t*(39)=−16.10, *p*<.001), respectively.

*ERP data:* After Carrus et al. (2013), we had four regions of interest (ROIs): right anterior (RA) (F6, FC4, F4, F2, FC2, FC6), left anterior (LA) (F3, F5, F1, FC3, FC5, FC1), right posterior (RP) (P6, PC4, P4, P2, PC2, PC6), and left posterior (LP) (P5, PC5, PC1, P3, P1, PC3). The following time windows were used for the analysis, based on previous literature (Astésano et al., 2004, Carrus et al., 2013, Eckstein and Friederici, 2005, Paulmann et al., 2012; Pinheiro, Vasconcelos, Dias, Arrais, & Gonçalves, 2015) and visual inspection of the ERPs: N1 (60–130 ms), P200 (100–200 ms), prosodic expectancy positivity (‘PEP’) (150–550 ms), P600 (500–800 ms), and P800 (850–1200 ms). Mean ERP amplitudes were calculated at each of the ROIs and individual time windows. As the ANOVA assumptions were met, a mixed factorial ANOVA was performed separately for each time window with the following five factors: *prosody* (statement, question), *note-probability* (high, low), *hemisphere* (left, right), *region* (anterior, posterior), and *musical training* (musicians, nonmusicians).

All statistical analyses were carried out using the IBM Statistical Package for the Social Sciences (IBM Corp. Released 2013. IBM SPSS Statistics for Windows, Version 22.0. Armonk, NY: IBM Corp.).
