# Supplementary material for: Musical training shapes neural responses to melodic and prosodic expectation
Source: Brain Res. 2016 Nov 1;1650:267–82. doi: 10.1016/j.brainres.2016.09.015 (PMC5069926; doi:10.1016/j.brainres.2016.09.015)
Supplement: Supplementary file 1 — Supplementary material [file mmc1.docx]

Language stimuli

1. The shelf has too many heavy books
2. We should buy him ten blue pens
3. Ricky has a job interview on Monday
4. Roger walked for more than three hours
5. Complaints are addressed to the store manager
6. My friend gave me a beautiful flower
7. They finally went on a relaxing vacation
8. My cousin ate a burger with fries
9. Maria smoked too many cigarettes last night
10. The leaders signed an agreement to trade
11. My dad runs five kilometers before eating
12. She is sending him a big package
13. Last night Martha visited an old friend
14. It has been raining nonstop this week
15. Last night John watched too many films
16. He replaced the wheels of his car
17. Yesterday Julia stopped sleeping with a blanket
18. My aunt now has another small child
19. Carole is baking a dark chocolate cake
20. I asked him for a big favour
21. I bought her an expensive leather jacket
22. He bought a car at the auction
23. They are running a marathon for charity
24. Michael is writing a long complaint letter
25. I will book a ticket to Paris
26. Each team has to nominate a leader
27. Tim borrowed a book from the library
28. They own a big house in London
29. I went to a nearby music festival
30. Mark is climbing a very high mountain
31. I teach English at a local school
32. Jenny is going to buy a bicycle
33. We walked across a long wooden bridge
34. This year the team won three matches
35. I cooked a meal for five guests
36. Several of the victims are receiving treatment
37. I think you should call a doctor
38. Terry is cooking a turkey for Christmas
39. Nicole is going to watch a film
40. Yesterday Max bumped into an old colleague
41. George is printing a document for work
42. Jason told me a very funny joke
43. I lost my keys in the car
44. Josh will speak at a professional seminar
45. Frank is baking bread for his family
46. Mary is calling her mum to talk
47. This morning Matt shot a big deer
48. Last night my cat caught a mouse
49. She moved to a very small village
50. A fireman saved a girl from dying
51. Katie is now peeling a fresh orange
52. Valerie is driving her car to work
53. They are throwing a party on Saturday
54. Paul is now playing his favorite game
55. He built a shed for his tools
56. She really likes telling stories to children
57. Kelly needs to talk to a lawyer
58. He should be serving the new customers
59. Tomorrow I have to post an urgent letter
60. Our offices are on the next floor
61. Julia drove her brand new car today
62. I was eating a really delicious pizza
63. Greg works at a restaurant on Sundays
64. They now rent a house in Washington
65. Eddy plays basketball every Friday with friends
66. The speaker was interrupted by a noise
67. She wanted to leave the room immediately
68. My father is working in his office
69. Emmanuel was a very talented rock singer
70. I will have some soup for lunch
71. The boxes contain many toys and games
72. Students like to study in the evenings
73. I tried so hard to learn Spanish
74. The car turned around in the road
75. They sat to eat at the table
76. This garden is full of young children
77. Travelling has helped to expand her horizons
78. Alicia goes to the library every day
79. I gave the lady my student card
80. Sam refused to sign the private documents
81. Michelle has a terrible sense of direction
82. Josh and Adam play football every afternoon
83. We have a big car waiting outside
84. He travels up north once a week
85. His jeans had holes in both knees
86. Shirley was driving along for several miles
87. He rang the doorbell more than once
88. We took the plants into the greenhouse
89. The kids were opening their birthday presents
90. Doreen lives in a very small house
91. Yesterday Terry found a ten pound note
92. Jamie tried hard to get this job
93. We need warm clothes for the winter
94. She can contact her tutor for advice
95. Mike hit his leg against a table
96. Children are naturally curious about the world
97. Dave is taking his exams next year
98. He should be examined by a doctor
99. Jane spent the whole summer picking strawberries

100. Bill is now walking down the street
