# Supplementary material for: Musical training shapes neural responses to melodic and prosodic expectation
Source: Brain Res. 2016 Nov 1;1650:267–82. doi: 10.1016/j.brainres.2016.09.015 (PMC5069926; doi:10.1016/j.brainres.2016.09.015)

Melodies: 1 - 10

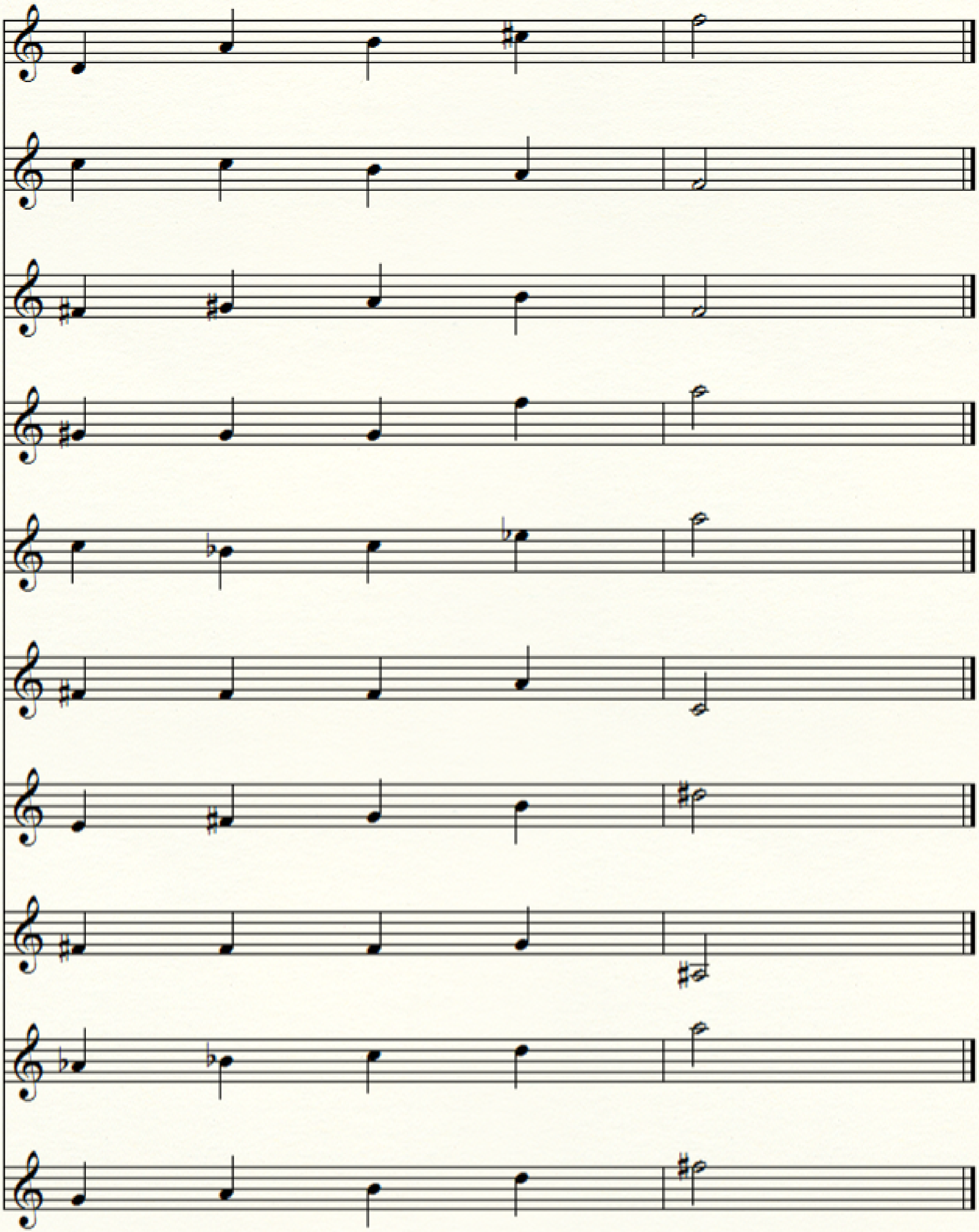

Melodies: 11 - 20

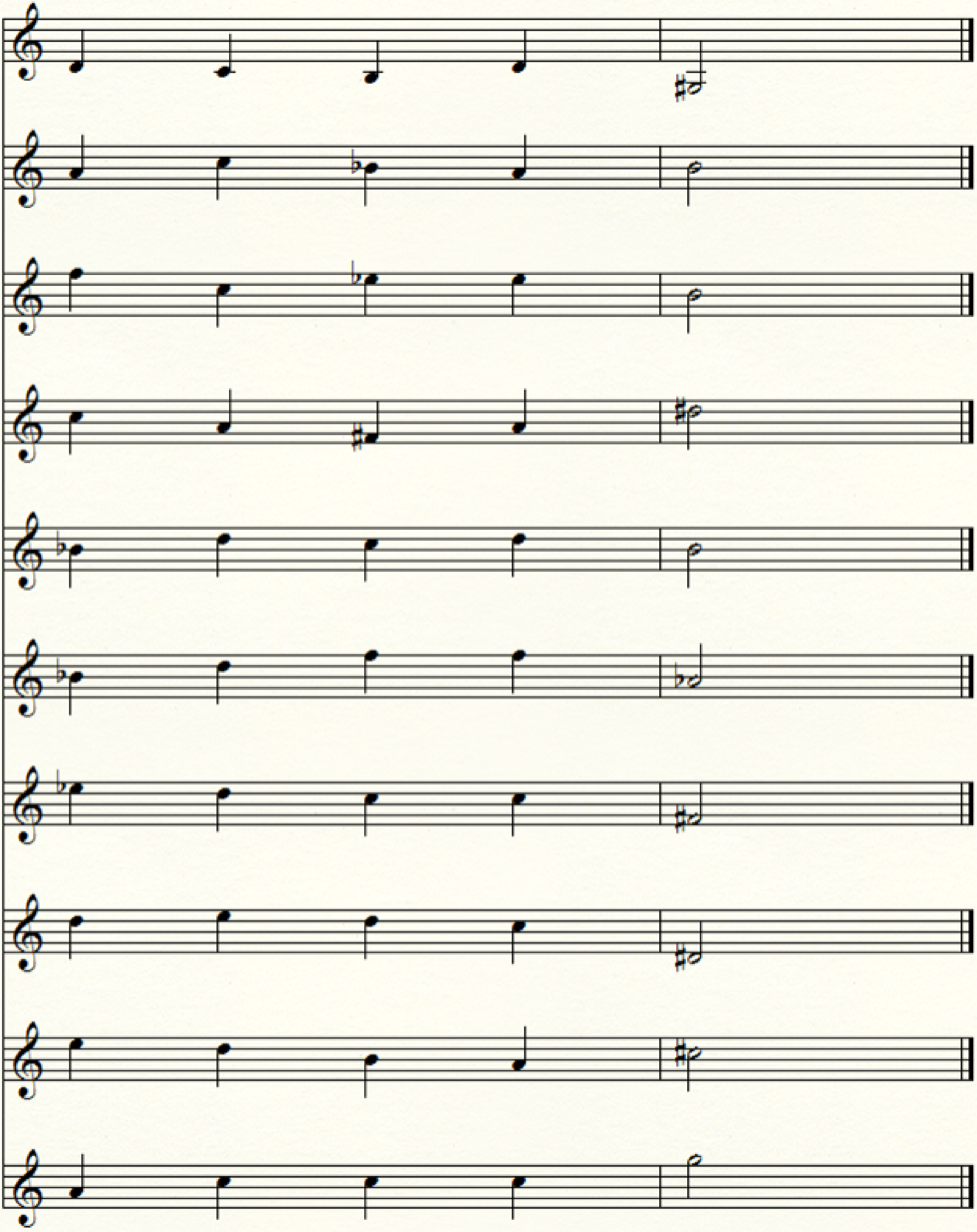

Melodies: 21 - 30

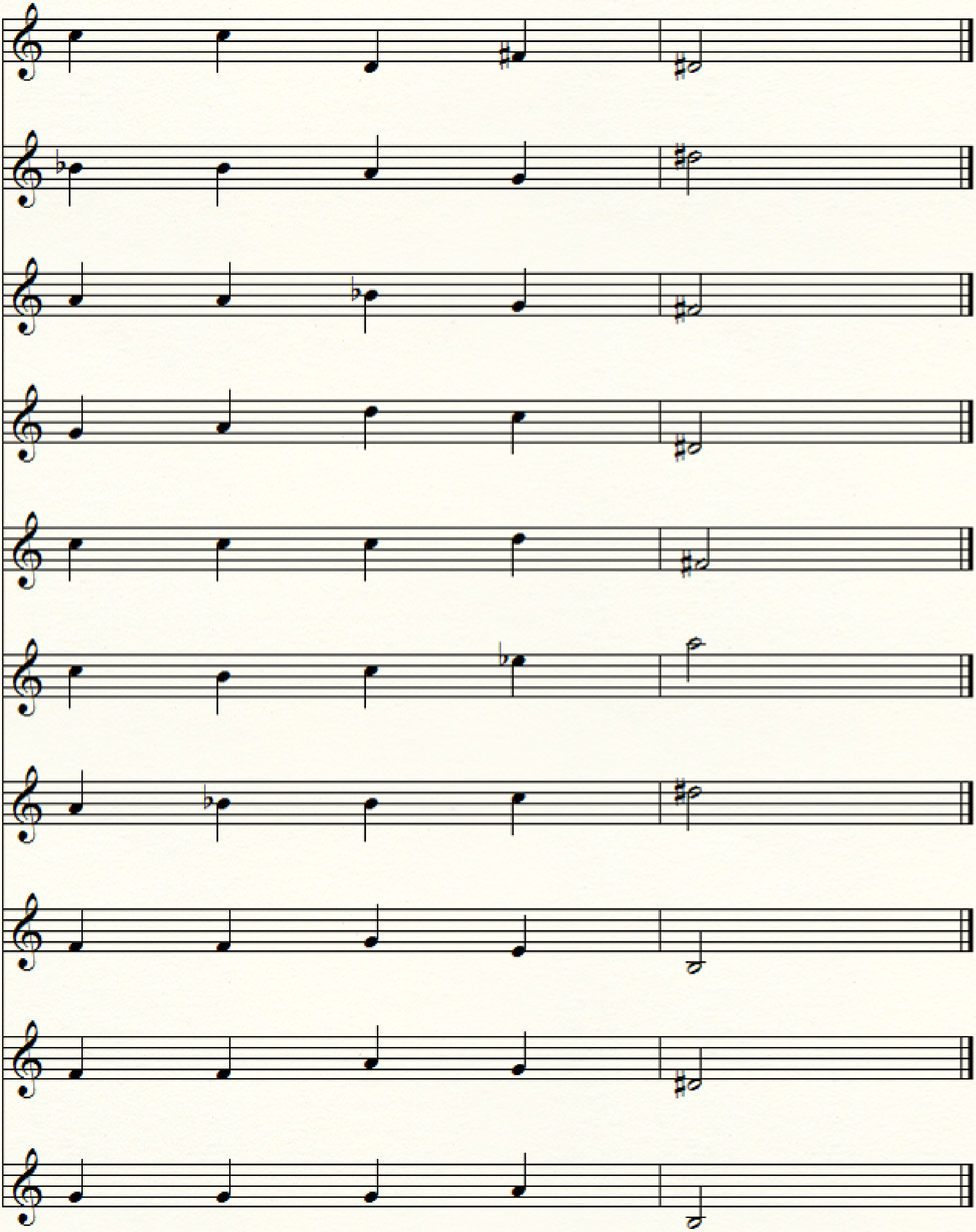

Melodies: 31 - 40

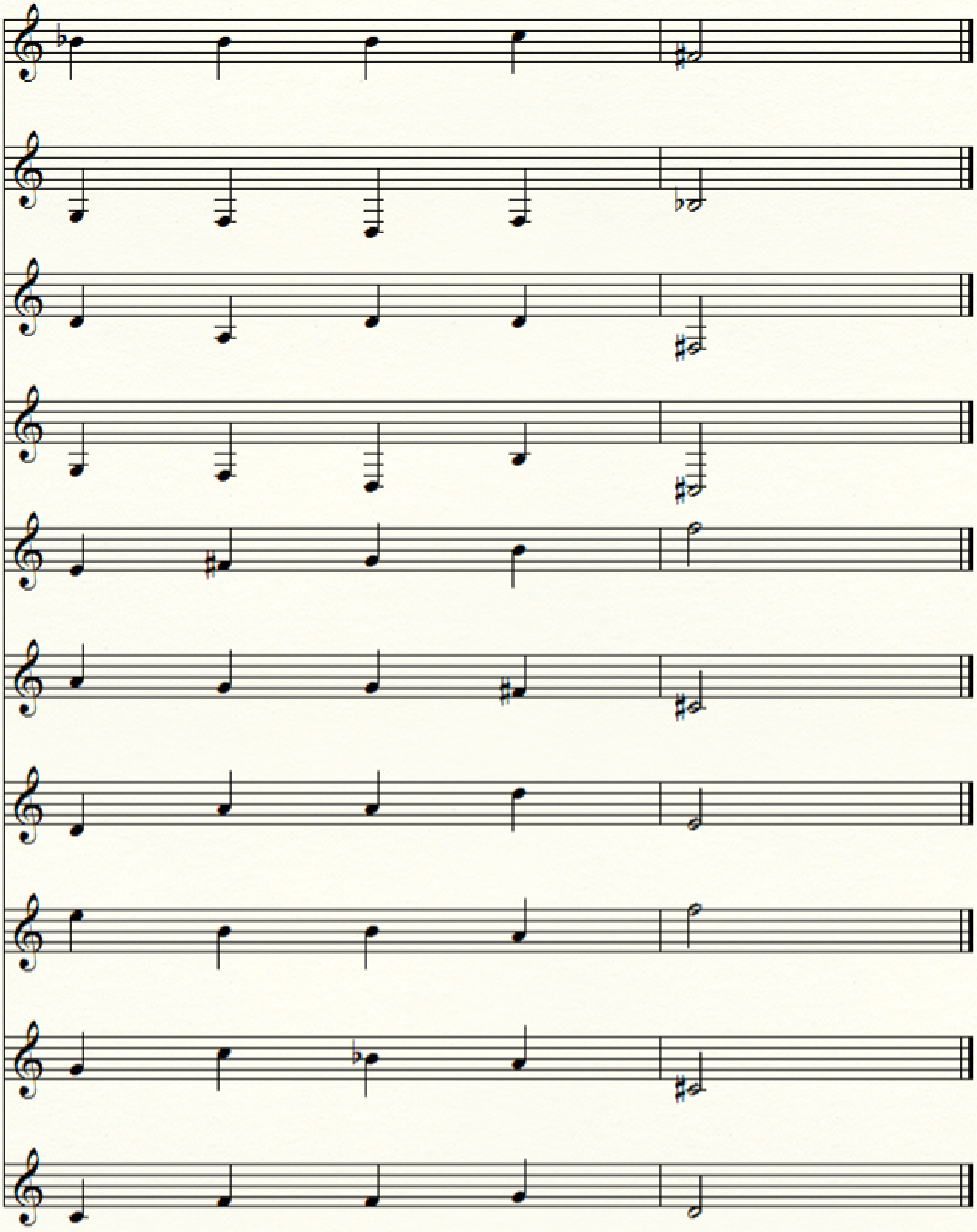

Melodies: 41 - 50

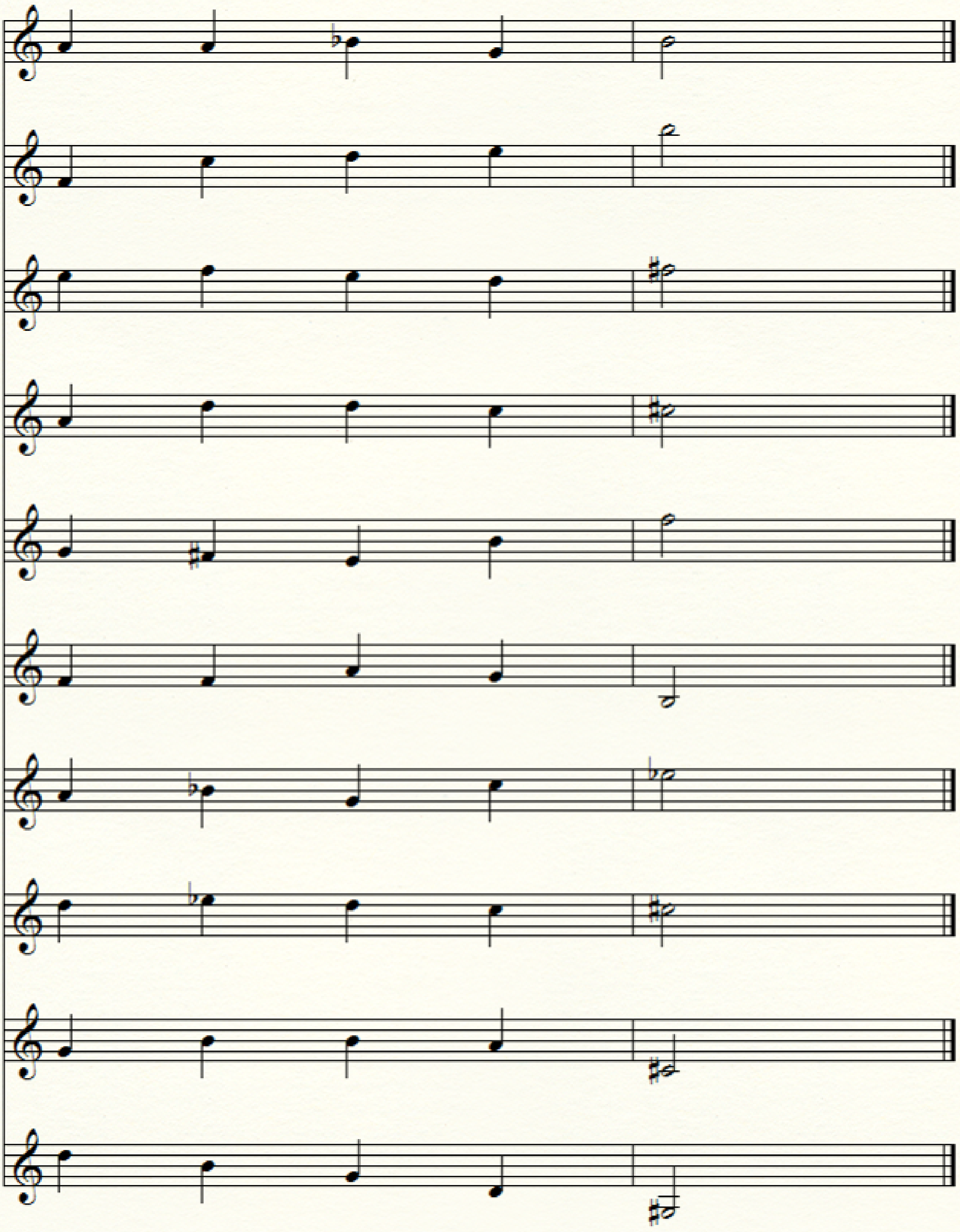

Melodies: 51 - 60

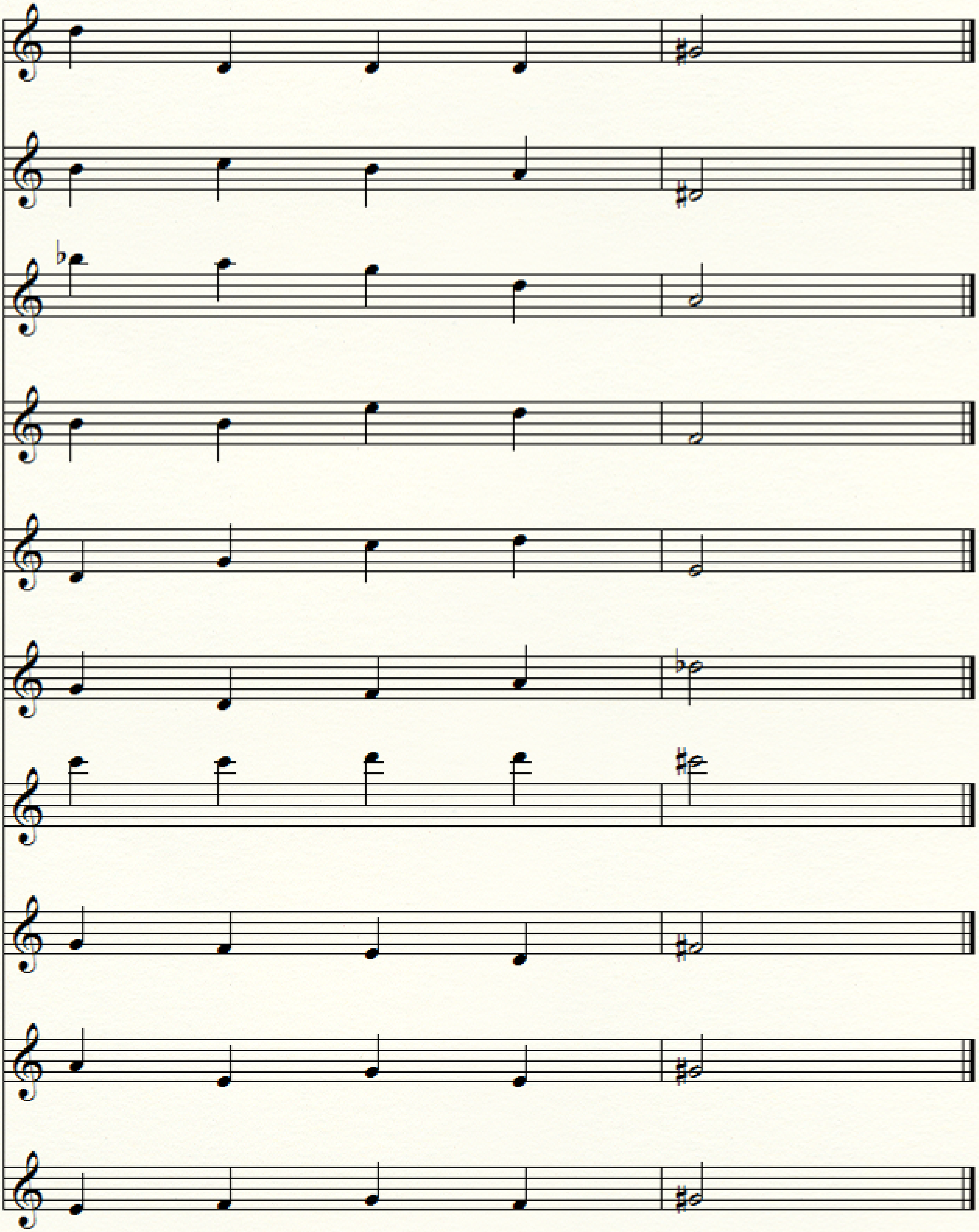

Melodies: 61 - 70

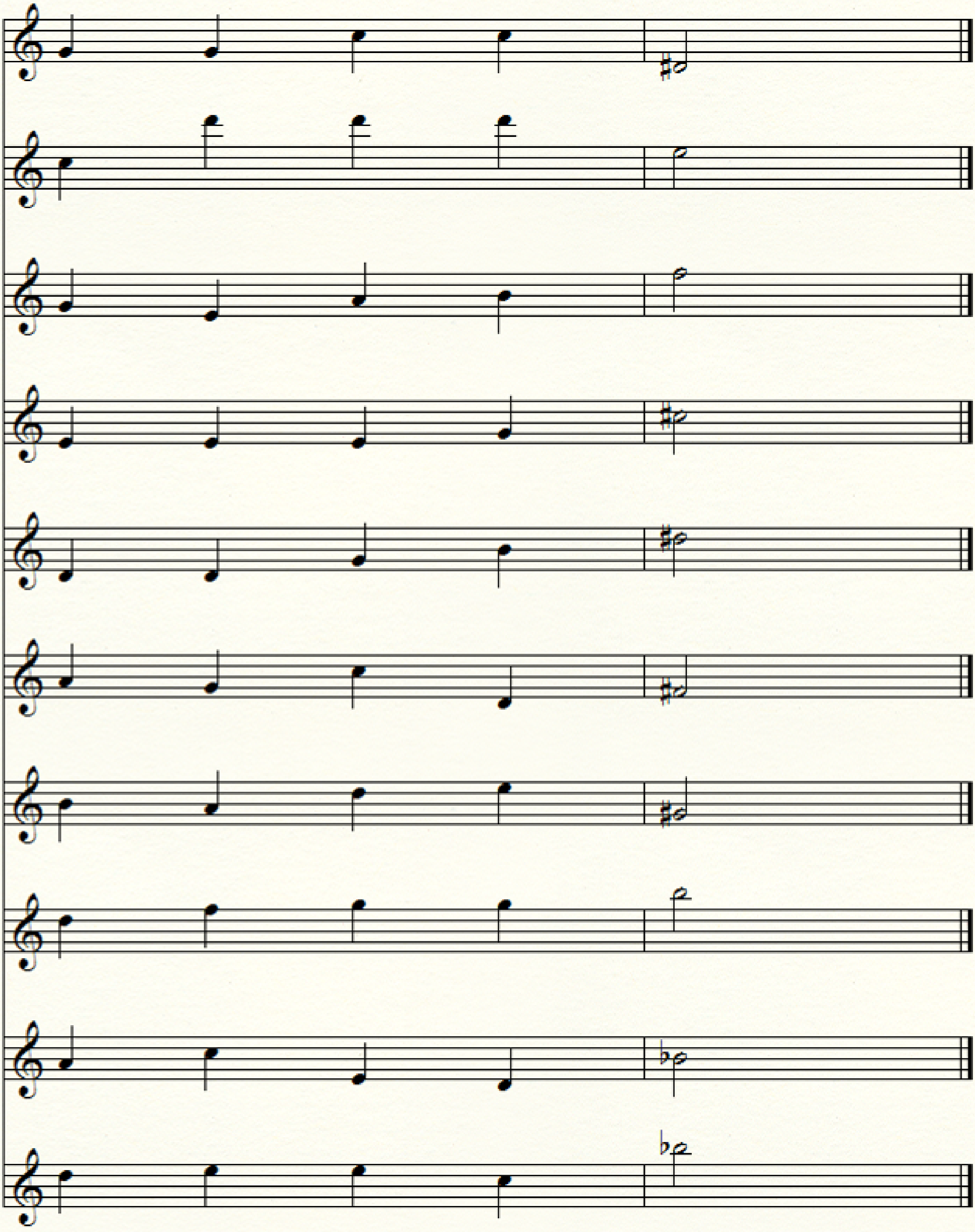

Melodies: 71 - 80

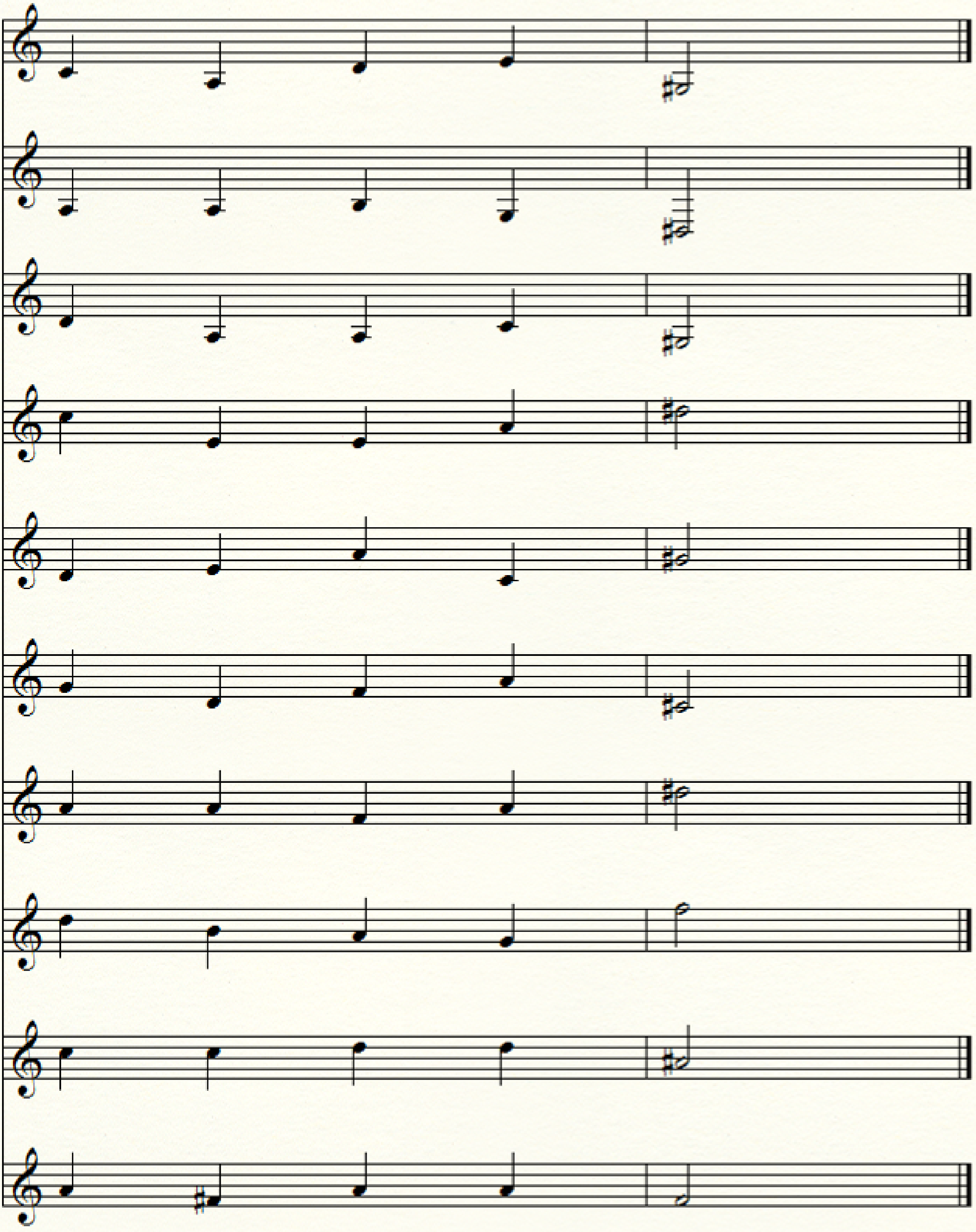

Melodies: 81 - 90

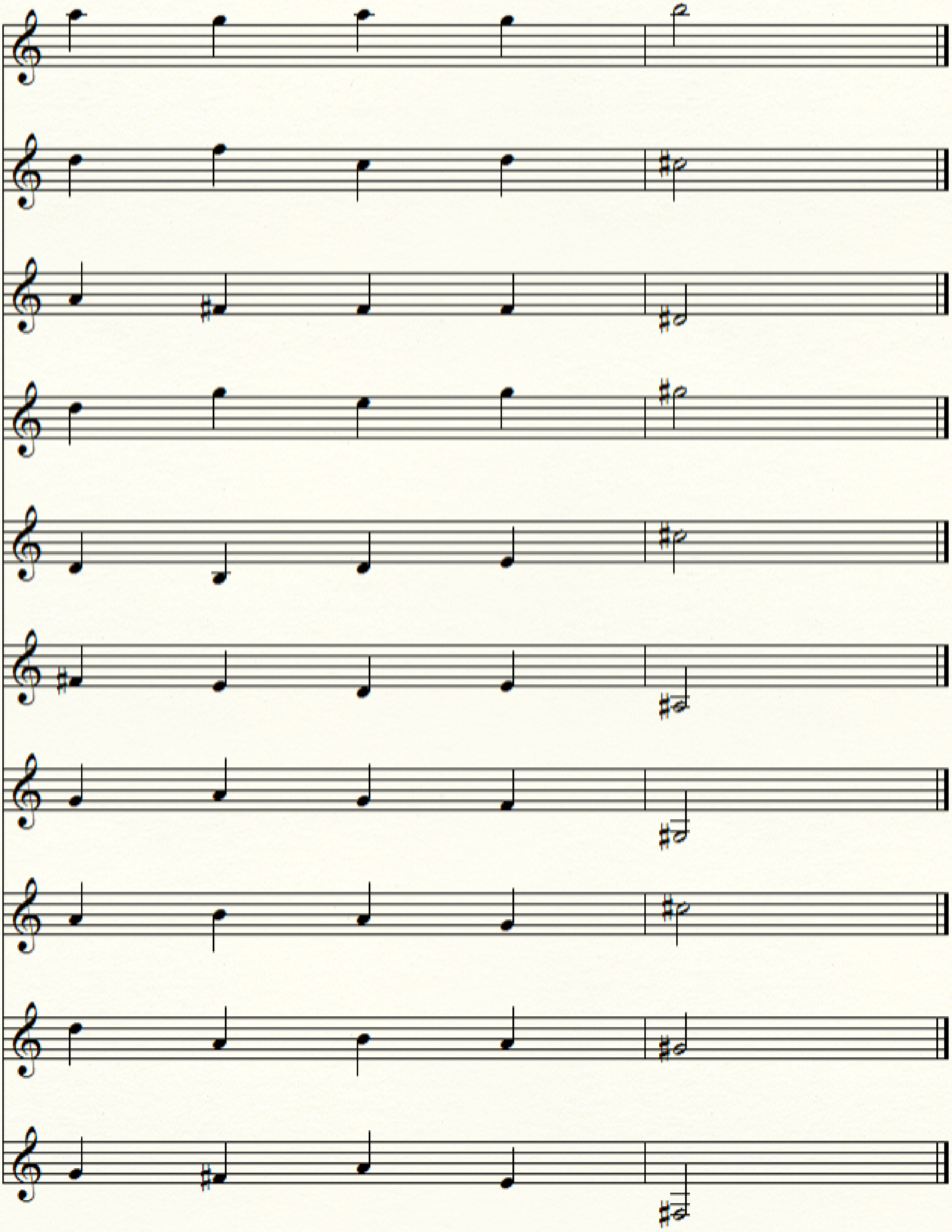

Melodies: 91 - 100

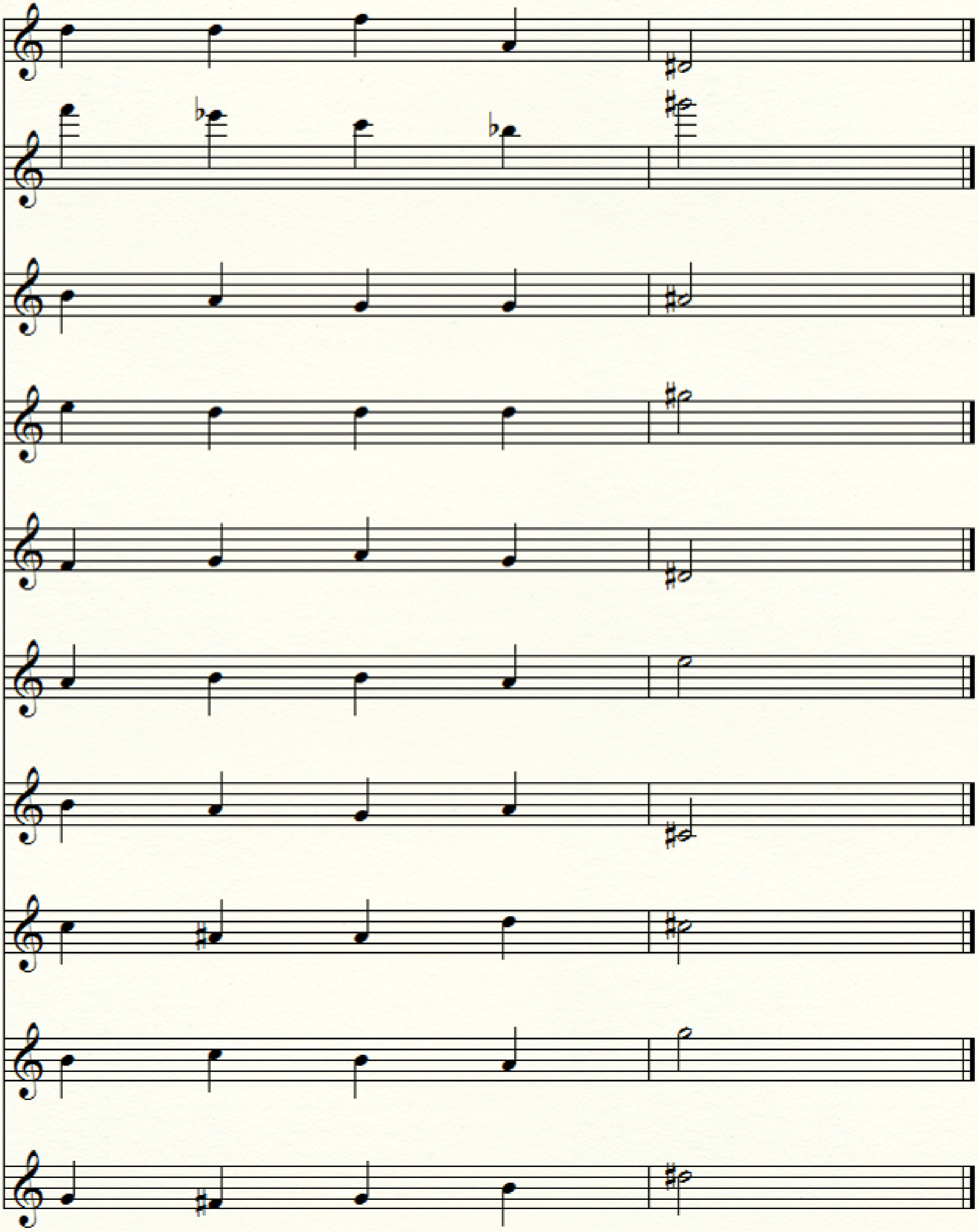

Supplement: Supplementary file 3 — Supplementary material [file mmc3.pdf]
