# Supplementary material for: Musical training shapes neural responses to melodic and prosodic expectation
Source: Brain Res. 2016 Nov 1;1650:267–82. doi: 10.1016/j.brainres.2016.09.015 (PMC5069926; doi:10.1016/j.brainres.2016.09.015)
Supplement: Supplementary file 4 — Supplementary material [file mmc4.pdf]

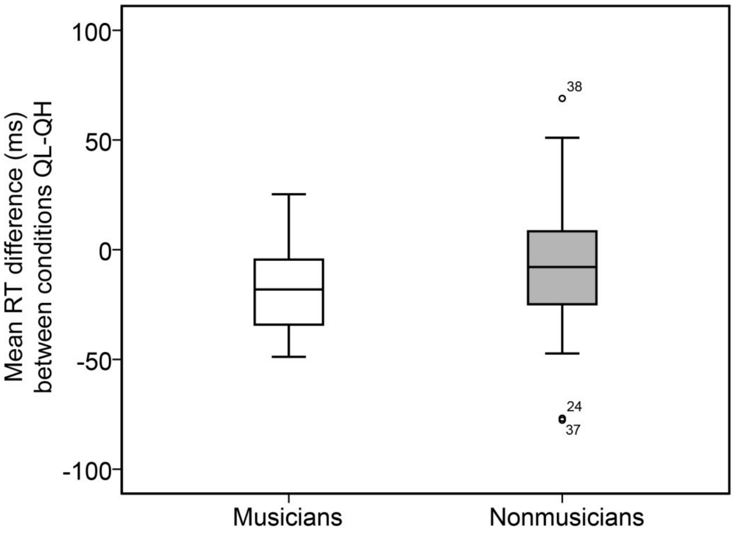

Figure – Boxplots with mean RT difference (ms) between conditions QL-QH, for musicians (white) and nonmusicians (gray).

Table

Dispersion measures (range, interquartile range, standard deviation, variance, minimum, and maximum) for mean RT differences (ms) between conditions QL-QH, for musicians and nonmusicians

	Musicians	Nonmusicians
Range	74.11	146.62
Interquartile range	32.91	42.81
Standard deviation	20.56	39.78
Variance	422.75	1582.182
Min	-48.83	-77.67
Max	25.28	68.95
